# Development of Thermophilic Tailor-Made Enzyme Mixtures for the Bioconversion of Agricultural and Forest Residues

**DOI:** 10.3389/fmicb.2016.00177

**Published:** 2016-02-16

**Authors:** Anthi Karnaouri, Leonidas Matsakas, Evangelos Topakas, Ulrika Rova, Paul Christakopoulos

**Affiliations:** ^1^Biochemical Process Engineering, Chemical Engineering, Department of Civil, Environmental and Natural Resources Engineering, Luleå University of TechnologyLuleå, Sweden; ^2^Biotechnology Laboratory, Department of Synthesis and Development of Industrial Processes, School of Chemical Engineering, National Technical University of AthensAthens, Greece

**Keywords:** enzyme mixture, thermostable enzymes, *Myceliophthora thermophila*, hydrolysis, experimental design

## Abstract

Even though the main components of all lignocellulosic feedstocks include cellulose, hemicellulose, as well as the protective lignin matrix, there are some differences in structure, such as in hardwoods and softwoods, which may influence the degradability of the materials. Under this view, various types of biomass might require a minimal set of enzymes that has to be tailor-made. Partially defined complex mixtures that are currently commercially used are not adapted to efficiently degrade different materials, so novel enzyme mixtures have to be customized. Development of these cocktails requires better knowledge about the specific activities involved, in order to optimize hydrolysis. The role of filamentous fungus *Myceliophthora thermophila* and its complete enzymatic repertoire for the bioconversion of complex carbohydrates has been widely proven. In this study, four core cellulases (*Mt*CBH7, *Mt*CBH6, *Mt*EG5, and *Mt*EG7), in the presence of other four “accessory” enzymes (mannanase, lytic polyssacharide monooxygenase *Mt*GH61, xylanase, *Mt*Fae1a) and β-glucosidase *Mt*BGL3, were tested as a nine-component cocktail against one model substrate (phosphoric acid swollen cellulose) and four hydrothermally pretreated natural substrates (wheat straw as an agricultural waste, birch, and spruce biomass, as forest residues). Synergistic interactions among different enzymes were determined using a suitable design of experiments methodology. The results suggest that for the hydrolysis of the pure substrate (PASC), high proportions of *Mt*EG7 are needed for efficient yields. *Mt*CBH7 and *Mt*EG7 are enzymes of major importance during the hydrolysis of pretreated wheat straw, while *Mt*CBH7 plays a crucial role in case of spruce. Cellobiohydrolases *Mt*CBH6 and *Mt*CBH7 act in combination and are key enzymes for the hydrolysis of the hardwood (birch). Optimum combinations were predicted from suitable statistical models which were able to further increase hydrolysis yields, suggesting that tailor-made enzyme mixtures targeted toward a particular residual biomass can help maximize hydrolysis yields. The present work demonstrates the change from “*one cocktail for all*” to “*tailor-made cocktails”* that are needed for the efficient saccharification of targeted feed stocks prior to the production of biobased products through the biorefinery concept.

## Introduction

Cellulose, the most abundant polysaccharide on Earth, is a remarkable pure organic polymeric component of plant material, consisting solely of 1,4-linked β-D-glucopyranose units held together in a giant straight chain molecule. Wood represents a composite material with cellulose as a major part combined in excellent form with lignin and hemicelluloses, creating a unique high-strength and durable material, and recently came again into focus as a renewable energy resource. In nature, a variety of microorganisms are known for producing a set of enzymes capable of degrading this insoluble polymer to soluble sugars, primarily cellobiose, and glucose. Enzymes involved in these processes are called cellulases and are consisting of at least three classes of enzymes, namely, *endogluganases* (EG), *cellobiohydrolases* (CBH), and β*-glucosidases* (BG). Apart from cellulases, an enzymatic system capable of oxidative cleavage is required for the cellulose degradation, consisting mainly of lytic polysaccharide monooxygenases (LPMOs) that increases the efficiency of cellulases (Horn et al., 2012). Cellulases can be used in the variety of applications within food, vine, animal feed, textile, and pulp and paper industry (Bhat, 2000). The application and interest in cellulases has particularly increased in recent years with the utilization of these enzymes, together with enzymes hydrolyzing hemicellulose (Rosgaard et al., 2007; Zhang et al., 2010) in the production of bioethanol from lignocellulose (Sun and Cheng, 2002). It has been predicted that a diverse range of plant biomass will be needed to satisfy the projected demands for advanced biofuels (Fargione et al., 2008), covering different types of lignocellulosic materials. Apart from biofuels, industrial bioproducts, chemicals, and materials that can be produced from the decomposition of biomass, play a key role in the so-called “biorefinery concept” for fostering a new bioindustry (Paster et al., 2003; Cherubini, 2010).

Forestry and agriculture residues are by nature heterogeneous in size, composition, structure, and properties (Taherzadeh and Karimi, 2008). Therefore, there are differences in the degradability of the materials (Kumar et al., 2009). Agricultural residues, such as wheat straw, have the advantage that in most cases they are easier to degrade in comparison with forest residues. This is mainly due to the lower lignin content (Kumar et al., 2009), but also due to the fact that cereals exhibit simpler cell organization with lower cell wall differentiation degree and fewer secondary structures observed (Biermann, 1997). Comparing the different types of woods, softwoods are generally more resistant to enzymatic hydrolysis compared to hardwoods, as the former contains higher lignin content (Taherzadeh and Karimi, 2008). In hardwoods and agricultural plants, xylan is the dominant hemicellulosic structure, whereas for softwoods, it is glucomannan, leading to the hypothesis that different types of biomass require a minimal set of enzymes that has to be tailor-made (Banerjee et al., 2010a), i.e., more xylanases for hardwoods or more mannanases for softwoods.

For the efficient hydrolysis of different types of lignocellulosic materials, novel enzyme mixtures have to be customized. Development of these cocktails requires better knowledge about the specific activities involved, in order to optimize hydrolysis. It would also be possible to supplement these enzyme mixtures with appropriate activities that could significantly enhance the hydrolytic potential over a range of substrates. In order to understand better the role of the individual enzymes and their synergistic interactions, the hydrolysis of wheat straw, one type of softwood (spruce) and one type of hardwood (birch) by a six component mixture at different stages was analyzed. All substrates had been pretreated to ensure an efficient enzymatic hydrolysis of cellulose by breaking down the shield formed by lignin and hemicellulose, while disrupting the crystalline structure and reducting the degree of polymerization of cellulose (Xiros et al., 2013). Different pretreatment technologies have varying effects on product yield and subsequent process steps (Wyman et al., 2005), as the overall enzyme performance is influenced by the accessibility and crystallinity of cellulose, as well as the residual lignin and hemicellulose. The enzymes used for the hydrolysis experiments were encoded by the filamentous fungus *Myceliophthora thermophila* (synonym *Sporotrichum thermophile*), a thermophilic filamentous fungus classified as an ascomycete, which constitutes an exceptionally powerful cellulolytic organism. The genome of this fungus possesses a large number of genes putatively encoding industrially important enzymes, such as carbohydrate-active enzymes, proteases, oxido-reductases, and lipases, while more than 200 sequences have been identified exclusively for plant cell-wall-degrading enzymes. These sequences encode a large number of glycoside hydrolases (GH) and polysaccharide lyases, covering the most of the recognized families (Karnaouri et al., 2014b). Most of these enzymes exhibit high thermostability. *M. thermophila*'s cellulases have been shown to remain stable for temperatures up to 60°C (Matsakas et al., 2015a). Thermophilic enzymes would have advantages in stability during the course of harsh process conditions and increased catalytic rates at higher temperatures, potentially reducing processing times, saving energy and improving fermentation yields in downstream processes. In this study, six enzymes encoded by *M. thermophila*'s genes were used to develop optimal mixtures using multi-component optimization protocols. The results suggest that the enzymes have distinct roles that can be partially redundant in the hydrolysis of different types of lignocellulosic materials. Optimal combinations were predicted from suitable statistical models that were able to further increase hydrolysis yields, suggesting that tailor-made enzyme mixtures targeted toward a particular residual biomass can help maximize hydrolysis yields.

## Materials and methods

### Substrates

Phosphoric acid swollen cellulose (PASC) was a generous offer from Prof. Mats Sandgren, Swedish University of Agricultural Sciences, Uppsala, Sweden. Spruce and birch were provided by SLU (Umeå, Sweden). Hydrothermal pretreatment of spruce and birch took place in SEKAB E-Technology AB (Örnsköldsvik, Sweden), while wheat straw (*Triticum aestivum* L.) was hydrothermally pretreated in a microwave digestion equipment at 195°C for 15 min as previously described for the pretreatment of sweet sorghum bagasse (Matsakas and Christakopoulos, 2013). Each of forest materials was hydrothermally pretreated with sulfur dioxide as a catalyst and was received as pretreated slurries of low pH (Matsakas et al., 2015b). All slurries were filtered and washed until the pH reached 5.0 prior of use. Carbohydrate and lignin compositional analysis was conducted with a two-stage sulfuric acid hydrolysis treatment, according to NREL procedure (Sluiter et al., 2010). Briefly, biomass samples were hydrolyzed by 72% (w/v) H_2_SO_4_ for 1 h at 30°C, followed by dilution to 4% (w/v) H_2_SO_4_ and autoclave at 121°C for 1 h. The acid insoluble lignin was determined gravimetrically and the sugars through HPLC equipped with and RI detector and an Aminex HPX-87P column (Bio-Rad Laboratories). Ash content of the forest residues was determined with incineration of the material at 550°C overnight and weighing of the residues (Sluiter et al., 2012).

The crystallinity index (CrI) in the pretreated materials was determined by X-ray diffraction (XRD) using a PANalytical Empyrean X-ray diffractometer, equipped with a PixCel3D detector and a graphite monochromator. CuKÜ1 radiation with λ = 1.540598 at 45 kV and 40 mA was used in the 2θ range 5–90° at a scanning speed of 0.026°/s. CrI was assessed with the XRD peak height method, developed by Segal and coworkers, that allows for rapid comparison between different cellulose samples. The equation used was:

$$\begin{array}{l} \text{CrI}=\left(I_{002}-I_{\text{AM}}\right)∕{\text{I}}_{002} \end{array}$$

where I_002_ and I_*AM*_ are the maximum and minimum intensity of diffraction at ~2θ = 22.4-22.6° and 2θ = 18.4-18.5°, respectively. I_002_represents the peak intensity of the crystalline and amorphous material, and I_AM_ represents the amorphous region only (Segal et al., 1962). Diffraction spectrum of Avicel PH-101 was used as a reference sample for crystalline cellulose.

### Enzymes

With the exception of xylanase Xyl6 and GH family 26 mannanase, all individual enzymes used in these experiments were produced in high cell density cultures of *Pichia pastoris*, in the controlled environment of bioreactors. EG *Mt*EG7 (GenBank ID:AEO58196.1), β-glucosidase *Mt*BGL3 (AEO58343.1), feruloyl-esterase *Mt*Fae1a (AEO62008.1), and LPMO *Mt*GH61 (AEO56542.1) genes from *M. thermophila* were cloned and heterologously expressed in the methylotrophic yeast as previously described (Moukouli et al., 2009; Dimarogona et al., 2012; Karnaouri et al., 2013, 2014a). Genes encoding EG *Mt*EG5 (AEO53769.1), as well as CBHs *Mt*CBH6 (AEO55787.1) and *Mt*CBH7 (AEO55544.1) were isolated from *M. thermophila*'s genome and cloned; *Escherichia coli One* Shot® Top10 (Invitrogen, USA) and Zero Blunt® PCR Cloning Kit (Invitrogen, USA) were used as the host-vector system, while *P. pastoris* host strain X33 and pPICZαC (Invitrogen, USA) were used for protein expression (unpublished data). Mannanase *Mt*Man26a has been cloned and expressed in *P. pastoris* from the research group of Biotechnology laboratory, School of Chemical Engineering, NTUA, Greece and was generously offered in purified form. Xylanase Xyl6 was a generous gift from Dyadic.

Cultivation of recombinant *P. pastoris* strains in bioreactors for the production of the enzymes was performed in the basal salts medium (BSM), supplemented by trace element solution PTM_1_, as described in the *Pichia* fermentation guidelines provided by Invitrogen (Invitrogen, *Pichia* Fermentation Process Guidelines). The PTM_1_ trace salt solution was also included in the glycerol- and methanol feeds during glycerol and methanol fed-batch phases. The only nitrogen source was ammonium hydroxide which was added as the pH was regulated. Cultivation started at 28°C, aeration was set at 4 vvm and agitation at 800 rpm. After 24 h of batch cultivation in glycerol medium (30 g/L initial concentration), the end of glycerol batch was indicated by a sharp increase in the dissolved oxygen (DO) tension. This stage was followed by a 5-h step of fed-batch glycerol one; during this step 50% w/v glycerol, with PTM_1_ salts was fed at an initial flow rate of 12 mL/h/L of culture medium and was reduced gradually until it was fully consumed. At the same time, temperature was reduced from 28 to 25°C and finally to 23°C and 2 mL of methanol were added manually in small aliquots with syringe. Total consumption of glycerol was again indicated by a spike in the DO. At the onset of methanol fed-batch phase, casamino acids solution was added at a final concentration of 3 g/L and then, a feed of 100% CH_3_OH, with PTM_1_ was initiated at a flow rate of 1.9 mL/h/L. The methanol consumption rate was monitored indirectly by stopping the feed and checking the *lag phase*, while increasing the methanol feed rate manually. After 8 h, feed rate was adjusted to a maximum of 5.5 mL/h/L and maintained for ~20 h, causing extracellular expression of the recombinant enzyme into the supernatant. Then, the temperature was decreased to 21°C and pure oxygen supply was set to maintain dissolved oxygen levels between 30 and 60%. Induction time lasted 160 h in total and ~700 mL of methanol were consumed.

At the end of the fermentation, the supernatants were filtrated and concentrated using a tangential flow filtration system with a 10 kDa-cutoff membrane (Pellicon XL Ultrafiltration Module Biomax 10 kDa, Millipore), buffer exchanged in dialysis tubing membrane with ten volumes of 100 mM phosphate—citrate buffer, pH 5.0, and then concentrated further to 20 mL. Concentrated desalted enzymes were rapidly purified by single-step immobilized metal ion affinity chromatography (IMAC), with a cobalt charged resin on an ÄKTA Prime Plus system, using 0–100 mM imidazole gradient, at a flow rate of 2 mL/min. Protein concentrations were determined using the bicinchoninic acid (BCA) protein assay microplate procedure (Pierce Chemical Co., Rockford, IL), according to the manufacturer's recommendations, using bovine serum albumin as standard (Smith et al., 1985). Enzyme activities of *Mt*EG7, *Mt*BGL3, *Mt*Fae1a, and *Mt*GH61 were assayed as described before (Moukouli et al., 2009; Dimarogona et al., 2012; Karnaouri et al., 2013, 2014a). CBH activity was tested against Avicel 5% (w/v) in 100 mM phosphate-citrate buffer pH = 5.0, after 24 h of incubation at 30°C and 200 rpm. Purity of the final protein preparations was determined by 12.5% SDS-PAGE electrophoresis.

### Enzymatic hydrolysis

Hydrolysis of PASC was performed using only the four core cellulolytic enzymes (*Mt*CBH7, *Mt*CBH6, *Mt*EG5, and *Mt*EG7) and β-glucosidase *Mt*BGL3. In case of lignocellulosic feedstocks, the addition of accessory enzymes was based on the structure and type of each material. In all experiments, *Mt*GH61 consisted 4% of the enzyme mixture, while gallic acid was added at 10 mM concentration, as electron donor. Xylanase consisted 3% of the enzyme mixture for birch (hardwood) and 2% for wheat straw and spruce (softwood). In case of spruce, *Mt*Man26a was added at 3% of the mixture. *Mt*Fae1a was used at 2% of the mixture for wheat straw and birch hydrolysis. Enzymatic reaction was performed in safe lock 2 mL volume microtubes. The surfactant octylphenol (ethyleneglycol)9,6 ether (Triton X-100) was added at all reactions with natural substrates at concentration 1 mg/mL, which is equivalent to a surfactant addition of 4% of the substrate dry matter. Enhancement of cellulose hydrolysis by adding surfactants to the hydrolysis mixture has been reported by several authors (Helle et al., 1993; Eriksson et al., 2002a). At first, Triton X-100 and substrate were added to the hydrolysis system before any other enzyme or chemical was added, followed by preincubation at 50°C for 1 h. Reactions were performed at 50°C, with 2.5% initial dry matter content when natural substrates were used and 0.25% in case of pure substrate (PASC). The enzymes were loaded at 1 mg/g substrate for PASC and 20 mg/g substrate for lignocellulosic materials. In all reactions, β-glucosidase was added in excess in order to prevent inhibition caused by cellobiose produced by the combined action of exo- and endo-1,4-β-glucanases. After 12 h of incubation, more *Mt*BGL3 was added, so as to ensure the effective release of glucose (Glc). All assays were replicated once, sampled twice and assayed twice for total reducing sugars (TRS) and Glc at 48 h of hydrolysis, for a total of four replicates of each mixture each time for each variable (TRS and Glc). TRS were measured with dinitrosalicylic acid colorimetric method (Miller, 1959) and Glc with Glucose Oxidase (GO) assay (Sigma). All reactions were performed with 1200 rpm agitation and contained 0.02% (w/v) sodium azide to prevent microbial contamination.

### Sugar analysis and modeling

An experimental design was set up for the four major cellulases *Mt*CBH7, *Mt*CBH6, *Mt*EG5, and *Mt*EG7, so as to achieve increased saccharification, whereas accessory enzymes (*Mt*Man26a, *Mt*GH61, xylanases, and *Mt*Fae1a) and *Mt*BGL3 were added at specific loadings, as mentioned above. More specifically the software Design Expert® 7.0.0 (Stat-Ease inc.) was employed where the algorithmically built *D-optimal* design was used in order to generate 20 experimental conditions (Table 1) where the four enzymes varied at specified levels (Table 2). The limits of these enzyme relative abundances were carefully chosen, in order to avoid not only working within a wide domain, as this may impact the reliability of predictions, but also limiting in a narrow domain, since extrapolation outside the borders is impossible. The lower and upper limits of each component were, therefore, determined combining data from the literature with rational considerations (Billard et al., 2012). In all the experimental combinations the summary of the enzymes was set to be equal to 1 (or 100%), so as in all the experimental conditions the same total amount to be used and only the proportion of each enzyme to vary. The same software was used in order to evaluate the results and determine the most appropriate model that would be used to fit the experimental data. The two models applied during this work were either the *quadratic* (Equation 1) or the *special cubic* (Equation 2):

(1) $$\begin{array}{l} y=\overset{4}{\underset{j=1}{\sum }}b_{j}\cdot x_{j}+\underset{1\leq j<k\leq 4}{\sum }b_{jk}\cdot x_{j}\cdot x_{k} \end{array}$$

(2) $$\begin{array}{l} y=\overset{4}{\underset{j=1}{\sum }}b_{j}\cdot x_{j}+\underset{1\leq j<k\leq 4}{\sum }b_{jk}\cdot x_{j}\cdot x_{k}+\\ \underset{1\leq j<k<m\leq 4}{\sum }b_{jkm}\cdot x_{j}\cdot x_{k}\cdot x_{m} \end{array}$$

where *y* is the response (either TRS or Glc, mg/mL), *b* are the coefficients that were estimated by the model and *x* are the variables of the model. Optimization of the mixture was also performed by the same software, where the option to maximize either TRS or Glc was set. At the same moment the concentration of the enzymes were set to be in the limits that they were chosen to vary (Table 2). The efficiency of the model was evaluated by calculating the *p*-value and *R*^2^. Theoretically predicted yields were verified with time-course experiments.

**Table 1:** **Experimental combinations used for the hydrolysis tests generated with ***D-optimal*** design (Design Expert® 7.0.0, Stat-Ease inc.)**.

**Number of reaction**	**Enzyme proportions**			
	***Mt*EG5**	***Mt*EG7**	***Mt*CBH6**	***Mt*CBH7**
1	0.06	0.07	0.65	0.21
2	0.20	0.05	0.53	0.22
3	0.01	0.40	0.20	0.39
4	0.12	0.33	0.24	0.31
5	0.20	0.40	0.20	0.20
6	0.01	0.05	0.47	0.47
7	0.01	0.08	0.21	0.70
8	0.09	0.19	0.52	0.20
9	0.03	0.19	0.35	0.44
10	0.01	0.40	0.20	0.39
11	0.01	0.24	0.21	0.54
12	0.01	0.05	0.47	0.47
13	0.01	0.33	0.46	0.20
14	0.20	0.40	0.20	0.20
15	0.20	0.21	0.20	0.38
16	0.13	0.05	0.20	0.62
17	0.01	0.33	0.46	0.20
18	0.06	0.07	0.65	0.21
19	0.11	0.24	0.37	0.28
20	0.20	0.05	0.37	0.38

*Each value represents the fraction (mg/mg) of the enzyme in the total enzyme mixture*.

**Table 2:** **The respective borders for all variables used the experimental design**.

	**Variable in model**	**Lower limit (%)**	**Upper limit (%)**
*Mt*EG5	χ_(1)_	1	20
*Mt*EG7	χ_(2)_	5	40
*Mt*CBH6	χ_(3)_	20	65
*Mt*CBH7	χ_(4)_	2	70

## Results

**Figure 1:**
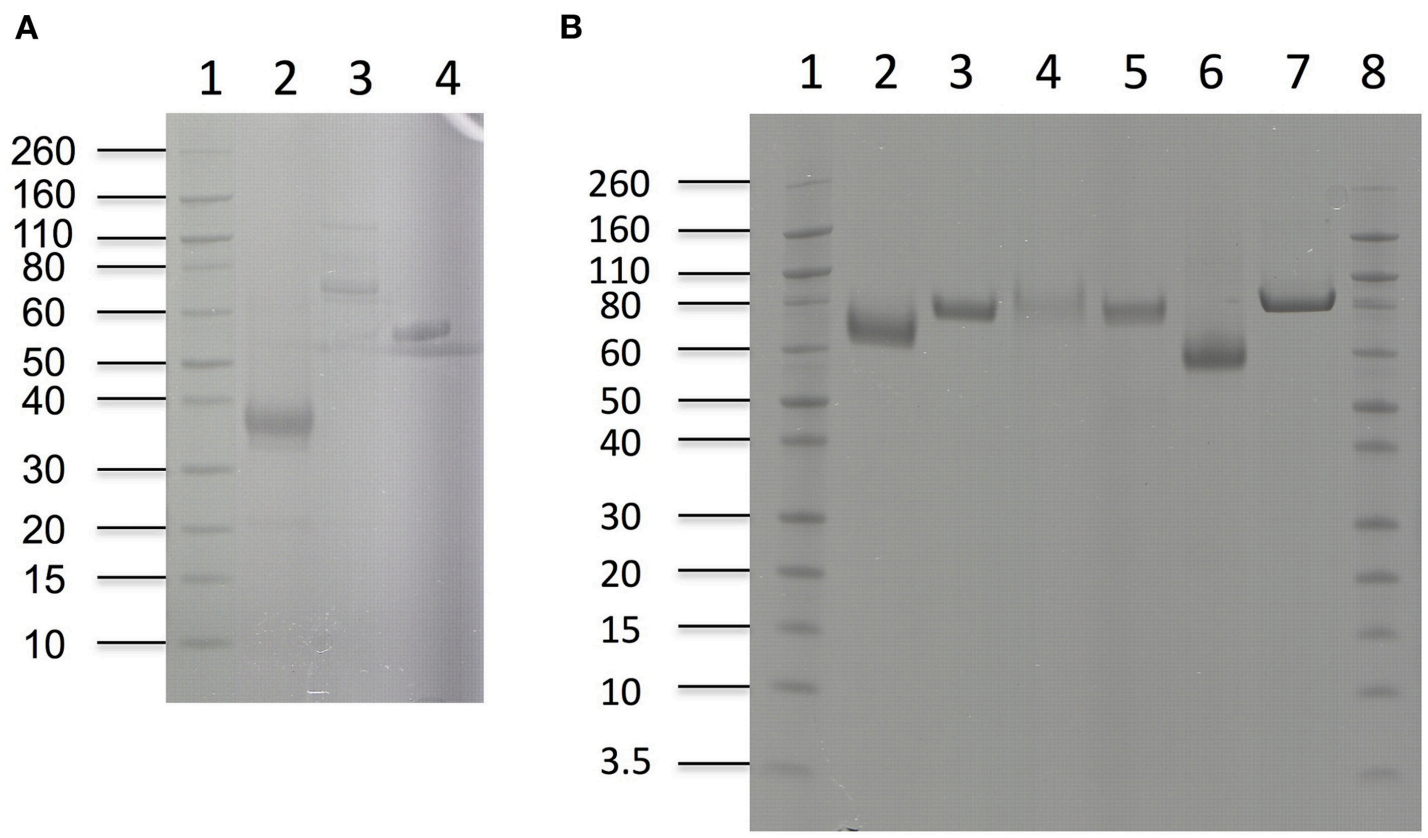
**SDS-PAGE of enzymes used in hydrolysis experiments**. **(A)** *Lanes 1*: Novex® sharp pre-stained protein marker, 2: *Mt*Fae1a, 3: *Mt*Man26a, 4: *Mt*Xyl **(B)** *Lanes 1*: Novex® sharp pre-stained protein marker, 2: *Mt*EG7a, 3: *Mt*EG5, 4: *Mt*CBH7, 5: *Mt*CBH6, 6: *Mt*GH61, *7: Mt*BGL3a.

**Figure 2:**
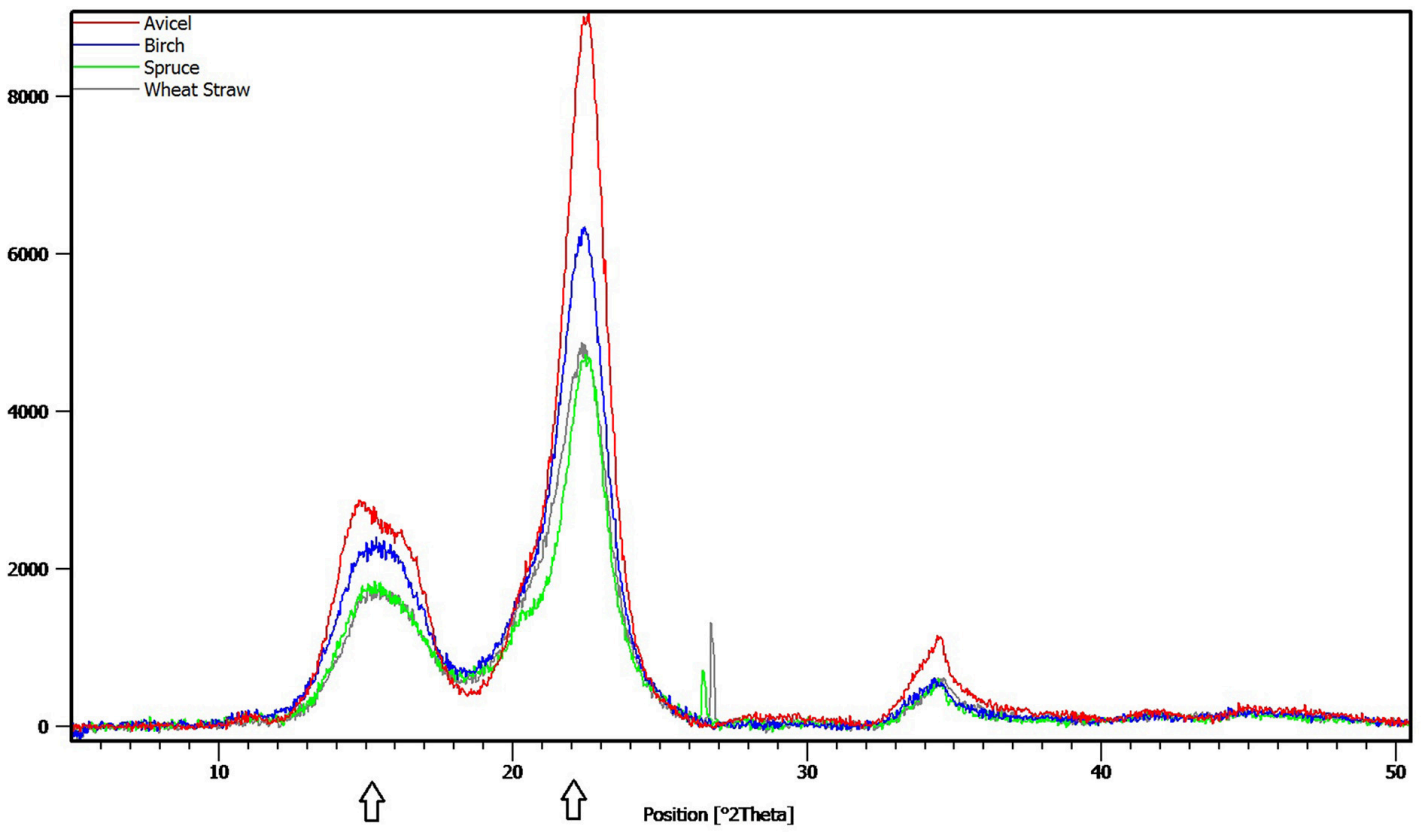
**X-ray diffraction spectra of Avicel PH-101 and pretreated wheat straw, spruce and birch samples**. The arrows indicate the peaks that were used to calculate the crystallinity index of the materials, as described in the Materials and Methods Section.

Nine different enzymes (Figure 1), all encoded by *M. thermophila*'s genome, representing the main cellulolytic and hemicellulolytic activities, were used for the construction of a multi-component cocktail and were tested against one “pure” cellulosic substrate (PASC) and three hydrothermally pretreated lignocellulolytic materials (wheat straw, spruce, birch; Table 3). The X-ray diffraction spectra of pretreated substrates were examined and compared, as shown in Figure 2. The CrI for all samples was calculated from the XRD data and revealed that birch showed the highest value, followed by wheat straw and spruce (Table 4). The relative proportions of a “core” set composed of four out of the nine enzymes (two EGs and two CBHs) were independently optimized for all substrates, while the accessory activities were added at specific loadings. TRS are expressed as a percentage of the total glucan content of the original feedstocks. Specific activities of the “core” enzyme set are given at Table 5. Table 6 shows the model prediction and the experimental results for TRS and Glc.

**Table 3:** **Compositional analysis for the pretreated materials used for hydrolysis in the present study**.

**Sample**	**Glucan**	**Xylan**	**Lignin**	**Ash**
Wheat straw	50.2	3.91	25.5	2.12
Spruce^*^	31.96	ND	46.69	0.36
Birch^*^	47.73	1.82	32.47	0.1

*ND, not detected*;

* *Matsakas et al. (2015b)*.

**Table 4:** **Crystallinity index (CrI) of Avicel PH-101 and pretreated lignocellulosic samples**.

**Sample**	**CrI**
Wheat straw	78.4
Spruce	77.3
Birch	88.2
Avicel PH-101	96.9

*The values were calculated after baseline subtraction of each spectrum*.

**Table 5:** **Specific activities of purified enzymes that constitute the “core” cellulase cocktail on solid model substrates**.

	***Mt*CBH6**	***Mt*CBH7**	*Mt*EG7^*^	***Mt*EG5**
Avicel	1.63	2.8	0.24	2.25
CMC	0.25	0.2	106	0.12

*The activities are expressed in μmol of glucose equivalents released per minute per mg enzyme. Substrate concentration: CMC 1% (w/v), Avicel 5% (w/v) for all enzymes, apart from MtEG7 that 1% (w/v) was used*.

* *Karnaouri et al. (2014a)*.

**Table 6:** **Composition and final Total Reducing Sugars (TRS) and glucose (Glc) yields for optimized mixtures**.

**Substrate**	***Mt*CBH7**	***Mt*CBH6**	***Mt*EG5**	***Mt*EG7**	**Mod. Pr**.	**Exp.data**
	**Optimized enzyme proportions (%)**				**TRS yield (%)**	
PASC	27.2	20.0	12.8	40.0	24.7	25 ± 0.7
Wheat straw	38.9	20.0	20	21.1	27.2	26.0 ± 1.1
Spruce	42.2	20.0	17.8	20.0	23.6	27.1 ± 0.9
Birch	24.8	35.6	20	19.6	8.8	7.4 ± 0.1
	**Optimized enzyme proportions (%)**				**Glc yield (%)**	
PASC	28.2	20.0	11.8	40.0	24.2	24 ± 0.9
Wheat straw	38.1	20.0	20.0	21.9	24.0	23.3 ± 0.3
Spruce	32.5	27.6	12.4	27.5	21.8	23.1 ± 0.8
Birch	20	39.1	20.0	20.9	6.7	6.9 ± 0.2

### Hydrolysis of PASC

**Figure 3:**
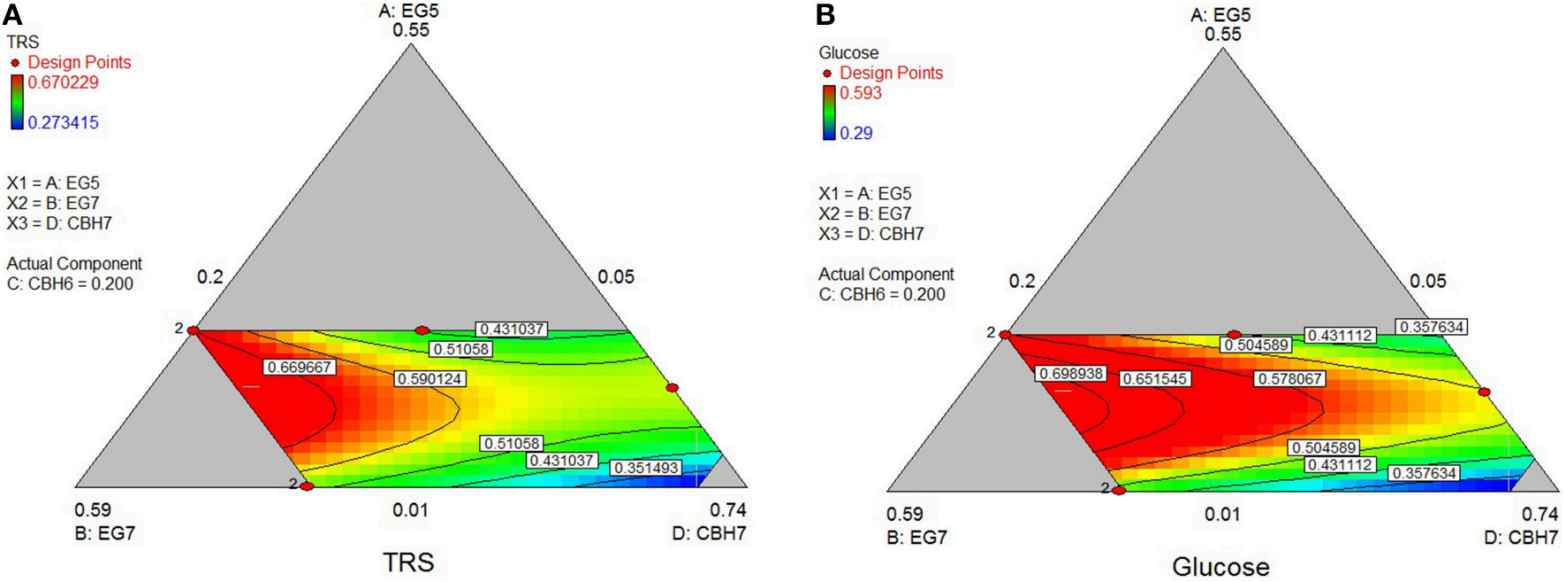
**Ternary plots showing predicted final total reducing sugars TRS (A) and Glc (B) yields from PASC hydrolysis, as a function of three out of four “core” enzymes (***Mt***CBH7, ***Mt***EG5, ***Mt***EG7) content**. For each plot, the forth enzyme (*Mt*CBH6) has been fixed to the proportion of the point resulting in the optimal TRS yield, as predicted by the model. Ternary plots with other enzyme combinations are shown at Supplementary Material.

The maximum yield of sugars and Glc released from the hydrolysis of *PASC* was calculated using the special cubic model (*p* = 0.0002, *R*^2^ = 0.9849 for the TRS and *p* = 0.0116, *R*^2^ = 0.9401 for glucose) and reached 0.69 and 0.58 mg/mL, respectively. This corresponded to 24.8% hydrolysis of the substrate and was achieved with high levels of *Mt*GH7 (40%) and MtCBH7 (27–28%; Table 6). Experimental data using the optimal ternary mixture were close to the predicted ones (25% hydrolysis of substrate). As illustrated in Figure 3, a decrease in *Mt*EG7 proportion results in a decrease in hydrolysis yields, even if *Mt*EG5 levels are higher, indicating the key role of GH7 EG for the reaction. Even though it cannot compensate for *Mt*EG7, *Mt*EG5 is also an important enzyme; as moving toward those points (conditions, Figure 3) where *Mt*EG7 and *Mt*CBH7 are in moderate levels and *Mt*EG5 in its lower limit proportion, the hydrolysis rate is very low.

### Hydrolysis of pretreated wheat straw

**Figure 4:**
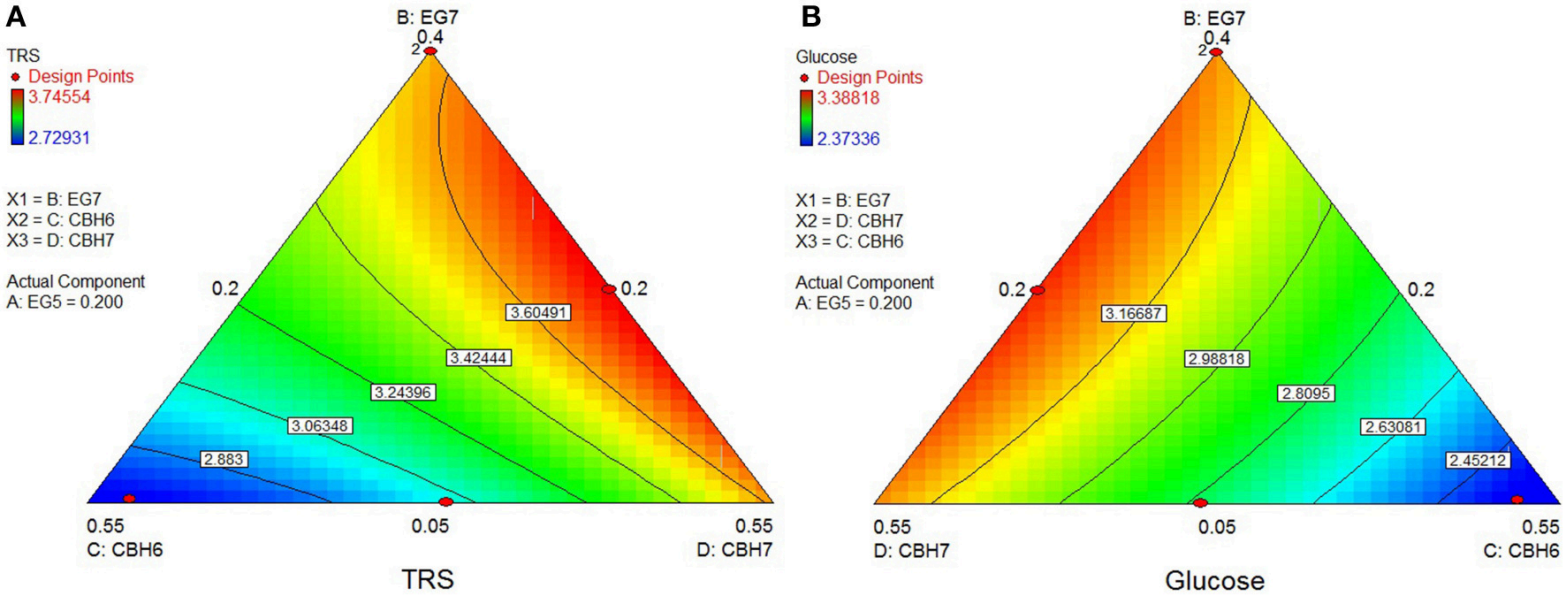
**Ternary plots showing predicted final total reducing sugars TRS (A) and Glc (B) yields from wheat straw hydrolysis, as a function of three out of four “core” enzymes (***Mt***CBH6, ***Mt***CBH7, ***Mt***EG7) content**. For each plot, the forth enzyme (*Mt*EG5) has been fixed to the proportion of the point resulting in the optimal TRS and Glc yield, as predicted by the model. Ternary plots with other enzyme combinations are shown at Supplementary Material.

The highest conversion of *wheat straw* was predicted using the quadratic model (*p* < 0.0001, *R*^2^ = 0.9548 for total reducing sugars and *p* = 0.0053, *R*^2^ = 0.8411 for glucose) and it was achieved with a ternary mixture of 38.9% *Mt*CBH7+20% *Mt*CBH6+20% *Mt*EG5+21.1% *Mt*EG7, where 27.2% of the substrate was converted. Experimental values of 48-h hydrolysis showed a slightly decreased yield (26.0 ± 0.4) in comparison to the predicted one. The ternary plots of Figure 4A show that, when the proportions of *Mt*EG7 are increasing or decreasing over a large range, a high final yield can be conserved. Same can be noticed also for *Mt*CBH7. Comparing hydrolysis yields obtained with high and low *Mt*CBH6/*Mt*CBH7 ratio it seems that when *Mt*CBH7 is in higher proportions, the yields are better. As the ratio decreases, the hydrolysis yield also follows the same tension, so *Mt*CBH6 does not compensate for *Mt*CBH7. Even when CBHs participate in low proportions (lower limits), there is some hydrolysis that can be attributed to the action of *Mt*EG7. Moving vertically toward lower *Mt*EG7 proportions, yields are maintained, so *Mt*CBH7 can compensate for *Mt*EG7 (at least partially). Similar assumptions may be made for ternary plot of Figure 4B concerning the Glc yield.

### Hydrolysis of pretreated forest materials (spruce, birch)

**Figure 5:**
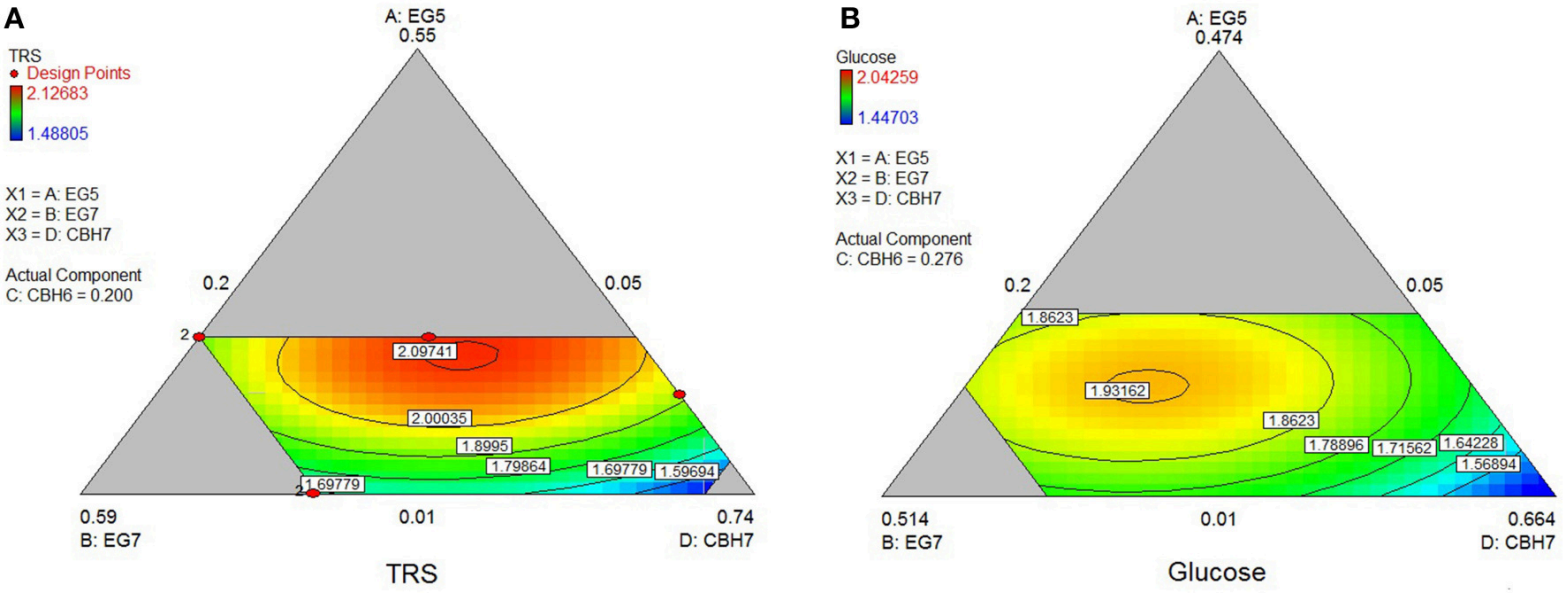
**Ternary plots showing predicted final total reducing sugars TRS (A) and Glc (B) yields from spruce hydrolysis, as a function of three out of four “core” enzymes (***Mt***CBH7, ***Mt***EG5, ***Mt***EG7) content**. For each plot, the forth enzyme (*Mt*CBH6) has been fixed to the proportion of the point resulting in the optimal TRS yield, as predicted by the model. Ternary plots with other enzyme combinations are shown at Supplementary Material.

The optimum TRS and Glc released from *spruce* hydrolysis was calculated using the quadratic model (*p* < 0.0001, *R*^2^ = 0.9463 for TRS and *p* = 0.0060, *R*^2^ = 0.8364 for glucose) and reached a hydrolysis level of 23.6%. Experimental values showed an increased yield, where 27.1% of the substrate was converted within hydrolysis. This result appears to be higher than the theoretically predicted one, but close to the reaction combination #20 that produced the highest amount of sugars (see Supplementary Material). *Mt*CBH7 constituted 42% of the optimal enzyme mixture and, together with *Mt*EG5, they are the key enzymes for maintaining the highest TRS yield, as highlighted by the optimal domains (Figure 5). *Mt*EG7 is also an enzyme with crucial role for the optimal Glc yield from spruce, as the optimal domain in ternary plots (Figure 5) is located where higher proportions of this enzyme are used in the enzymatic reaction mixture.

**Figure 6:**
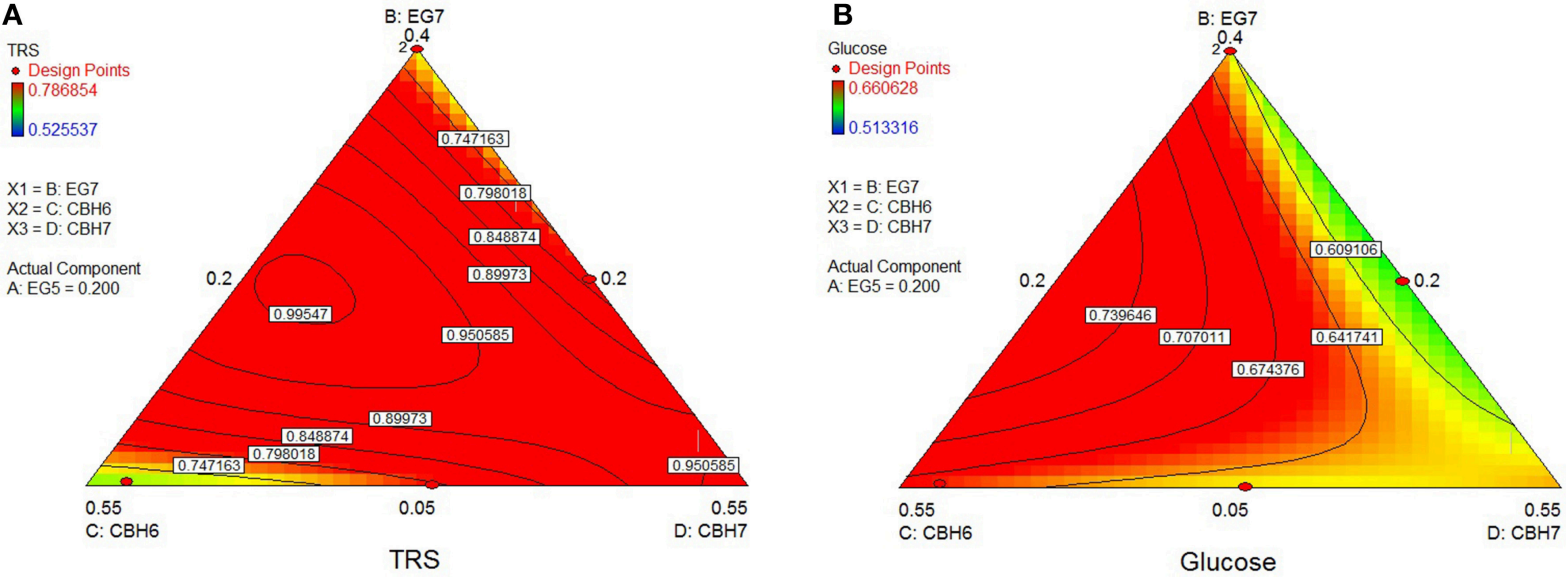
**Ternary plots showing predicted final total reducing sugars TRS (A) and Glc (B) yields from birch hydrolysis, as a function of three out of four “core” enzymes (***Mt***CBH6, ***Mt***CBH7, ***Mt***EG7) content**. For each plot, the forth enzyme (*Mt*EG5) has been fixed to the proportion of the point resulting in the optimal TRS and Glc yield, as predicted by the model. Ternary plots with other enzyme combinations are shown at Supplementary Material.

The rigid structure of *birch* hampered the efficient hydrolysis of its cellulolytic component, the extent of which reached only 7.4%. Optimum TRS and Glc release was calculated using the special cubic model (*p* < 0.0001, *R*^2^ = 0.9981 for TRS and *p* = 0.0008, *R*^2^ = 0.9764 for glucose) and achieved with a ternary mixture of 24.8% *Mt*CBH7+35.6% *Mt*CBH6+20% *Mt*EG5 + 19.6% *Mt*EG7. As shown at the ternary plots in Figure 6, the location of the optimal domain indicates that also *Mt*EG7 and *Mt*EG5 are important for efficient hydrolysis.

### Time course hydrolysis experiments

**Figure 7:**
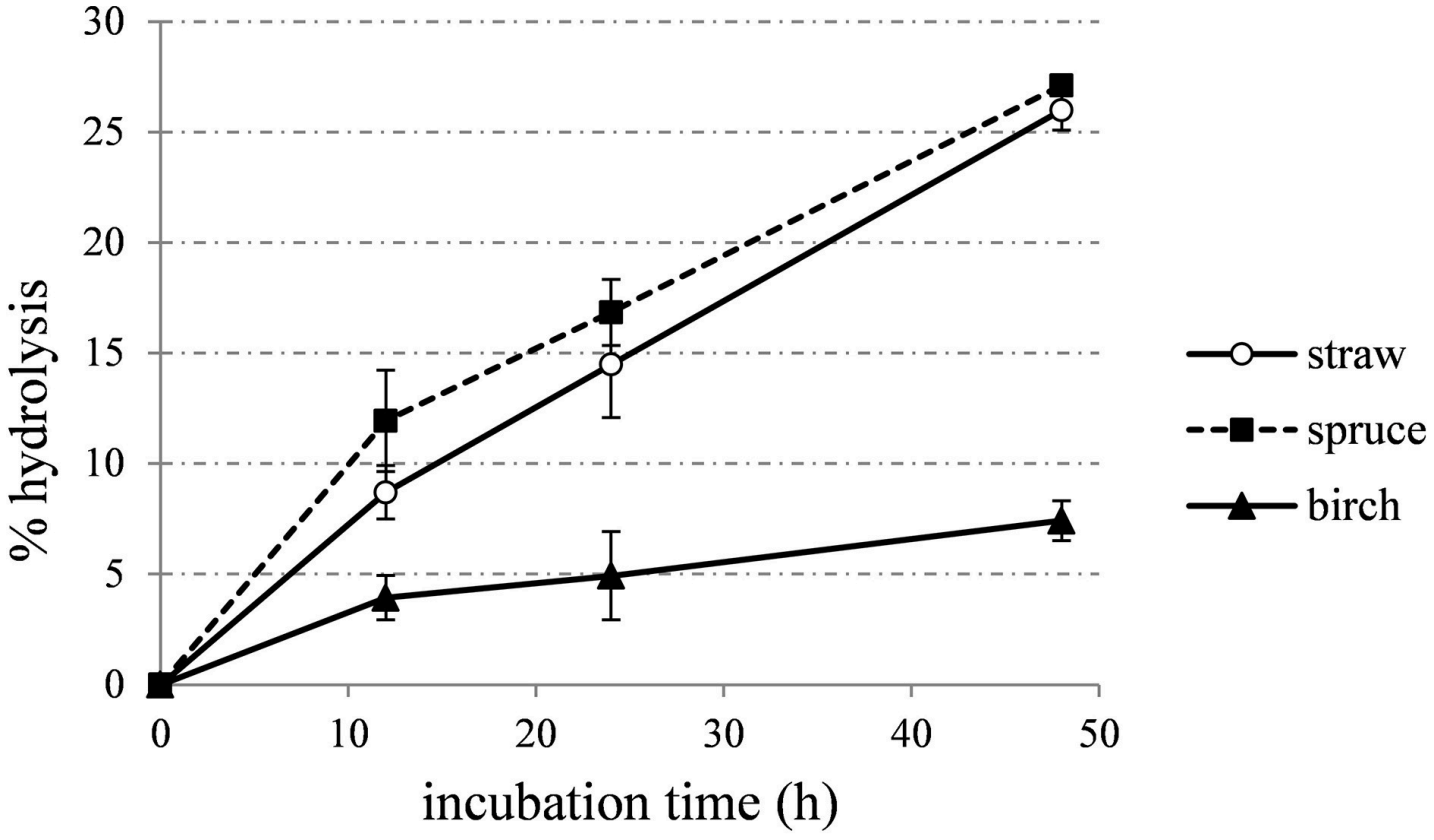
**Time course hydrolysis of wheat straw, spruce, and birch, using the enzyme combinations that were predicted to lead to the highest TRS production**.

The optimal enzyme combinations predicted targeting the maximum sugars yield for each material, were also tested in a time-course hydrolysis experiment. Enzyme mixtures for wheat straw, spruce and birch displayed activity of 12.01, 13.07, and 12.64 FPU/g of substrate respectively. During first 12 h, hydrolysis rate is higher than later stage of reaction, thus leading to decreased yields and long process times (Figure 7).

### Accessory enzymes and non-ionic surfactant effect in enzymatic hydrolysis

**Figure 8:**
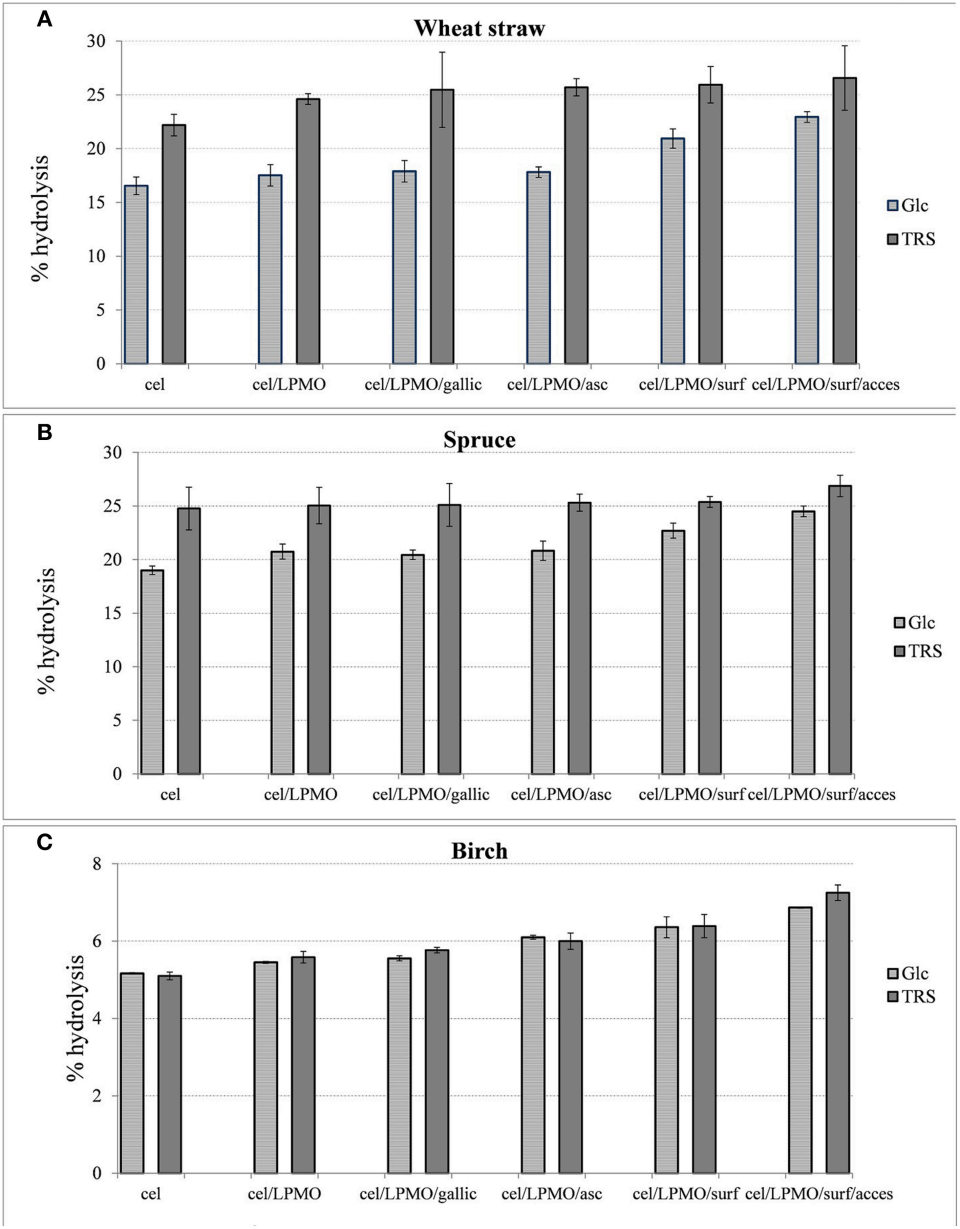
**The percentage increase after 48 h hydrolysis, with cellulases (***Mt***EG7, ***Mt***EG5, ***Mt***CBH6, ***Mt***CBH7, ***Mt***BGL3), of steam pretreated wheat straw (A), spruce (B), and birch (C) after the addition of ***Mt***GH61, reducing agents (gallic acid, ascorbic acid), surfactant (Triton X-100), and accessory enzymes (***wheat straw***: Xyl6, ***Mt***Fae1a, ***Mt***Man26a, ***spruce***: Xyl6, ***Mt***Man26a, ***birch***: Xyl6, ***Mt***Fae1a)**.

In order to evaluate the effect of accessory enzymes and surfactants in the performance of optimal reaction mixtures for each lignocellulosic material, differential conditions hydrolysis experiments were conducted, as illustrated in Figure 8. Supplementation of the enzyme mixture with 4% *Mt*GH61 resulted in 5–6% increase of Glc yield. The greater boosting effect of LPMO was observed on the wheat straw followed by hardwood and softwood. There was no significant boosting effect when gallic or ascorbic acid was used as an exogenous synthetic reducing agent. Addition of Triton-X-100 resulted in 17% increase of wheat straw and 5–7% of spruce and birch overall biomass saccharification. When the reaction was supplemented with accessory enzymes (xylanase, mannanase in case of spruce and feruloyl esterase in case of birch and wheat straw), the yields increased by 9% for wheat straw, 13% for spruce, and 8% for birch. The overall increase of hydrolysis levels, attributed to the whole of the additional components (*Mt*GH61, surfactant and accessory enzymes) reached 38% for wheat straw, 28% for spruce, and 32% for birch, referring to the amount of Glc released.

## Discussion

The performance of a cellulolytic cocktail against a model cellulosic substrate, such as Avicel or PASC is completely different from the one against natural lignocellulosic materials (Kabel et al., 2006; Berlin et al., 2007). In this study, the hydrolysis yields reached 25% against PASC when loaded at 1 mg/g of substrate, while in case of lignocellulosic substrates 20 mg/g of substrate were used to achieve 26% conversion of sugars in wheat straw, 27% in spruce, and 7.4% in birch. PASC is considered to be a representative of amorphous cellulose, with the macromolecular structure of cellulose as a moiety of fibers maintained (Zhang et al., 2006). In literature, optimization experiments of cellulase expression levels for efficient cellulose degradation have shown that high levels of EG expression are important for efficient hydrolytic activity of culture supernatant against amorphous cellulose such as PASC (Yamada et al., 2010) and it has been suggested that these substrates could almost be total degraded into Glc only by EG and BGL activity (Den Haan et al., 2007). EG5 acts as a processive enzyme by not only cleaving cellulose internally and also releasing soluble oligosaccharides from chain ends before detaching from the polysaccharide. Therefore, its activity results in alteration of the surface properties of the fibrils, while *Mt*EG7 mode of action enables digestion exclusively within the bulk of the amorphous cellulose. CBHs display synergy with EGs (Andersen et al., 2008), acting against small microcrystalline substructures, present within the relatively amorphous bulk of PASC.

In case of wheat straw hydrolysis, CBHs hold a key role for high sugar yields. So far, CBHs are known to be important for cellulose hydrolysis (Teeri, 1997). In addition, literature data have shown that higher CBH7/CBH6 ratios are more beneficial for hydrolysis of steam-exploded wheat straw than lower ones (Rosgaard et al., 2007). EGs are also of major importance for the efficient hydrolysis of pretreated wheat straw, catalyzing the initial attack on the amorphous regions of cellulose chains and the gradual reduction of the average chain length of these polysaccharides. Billard et al. (2012) showed that the optimum yield is conserved over a range from about 13 to 23% EG7. Szijarto et al. (2011) identified EG2 (Cel5a) as a key component for the liquefaction of pretreated wheat straw, while *Mt*EG7 has also been proved to liquefy efficiently high-consistency lignocellulosic biomass by decreasing significantly the viscosity of the slurry in the first stage of reaction, underlining the crucial role of this enzyme for hydrolysis (Karnaouri et al., 2014a). The crucial role of these enzymes is highlighted in the results of the present study. Synergistic interactions between CBH I and EG have also been proven to hold a key role in hydrolysis of steam pretreated spruce, mainly caused by EG facilitating the processive hydrolytic movement of CBH I (Eriksson et al., 2002b). Regarding birch hydrolysis, CBHs consist the main component of the reaction mixture producing the highest yields, with *Mt*CBH7 as the dominant factor. CBHs may be inhibited by xylan and xylan-fragments produced during the hydrolysis. Birch is a hardwood with its main hemicellulolytic component to be glucuronoxylan, so the addition of efficient proportion of xylanolytic enzymes is of great importance to maintain an optimal yield and eliminate the inhibitory effect that hampers the activity of CBHs. In this study, xylanase was used at a low proportion of 3%. Inhibition state is also illustrated at Figure 7, where the hydrolysis rate drops rapidly after first 12 h of incubation.

Time course hydrolysis experiments against the different substrates showed that the hydrolysis rate is higher during the first 12 h of incubation, but seems to decrease in later stages due to the recalcitrance of the substrates. The forest materials display a more rigid structure and higher lignin content than agricultural like wheat straw, so they usually are more resistant toward deconstruction. Along the axial axes, monocots (such as wheat straw) have a different cell distribution than that observed in dicotyledonous angiosperms and gymnosperms. Even after pretreatment, which traditionally targets to disruption of microfibril structure, reduction of CrI and removal of hemicellulose/lignin fractions, remaining cellulosic, and non-cellulosic components exert significant restraints on hydrolysis. Cellulose contains crystalline and amorphous regions, and crystallinity is one of the most important measurable properties of cellulose affecting its enzymatic digestibility (Lee et al., 1983; Mittal et al., 2011). Amorphous or completely disordered cellulose is hydrolyzed at a much faster rate than partially crystalline cellulose (Fan et al., 1980; Hall et al., 2010), thus rendering the degree of crystallinity an important factor for the determination of the enzymatic digestibility of a cellulose sample. In this study, the CrI of the pretreated substrates was evaluated with the XRD peak height method, one of the most widely used methods for the determination of CrI, due to its ease of use. This method produces higher values than the other methods reported in the literature, but can be used as a “time-saving empirical measure of relative crystallinity” (Park et al., 2010). The relatively higher CrI value of birch substrate implied that there was a relatively higher proportion of crystalline cellulose in the samples in comparison to wheat straw and spruce and may explain differences in performance of cellulases and observed hydrolysis rates. Pretreatment severity factor value was 4.08 for spruce, 3.35 for birch, and 3.94 for wheat straw (Matsakas and Christakopoulos, 2013; Matsakas et al., 2015b), thus revealing that pretreatment conditions were more harsh for spruce than for birch, giving a possible explanation for the different values of CrI. The pretreatment of birch had little effect on cellulose crystallinity, partially reasoning for the low hydrolysis yields. Other substrate properties, such as the degree of polymerization, paracrystalline regions, the available surface area, and the pore size have to be taken into consideration. Enzyme accessibility is affected by crystallinity but also by cellulose anatomy and the lignin and hemicellulose content/distribution. These components restrict the access of cellulolytic enzymes; as a result, enzyme mixtures with similar cellulose activity may show differences in performance on different lignocellulosic materials (Berlin et al., 2006a,b).

The addition of surfactants increases hydrolysis yields and the rate of enzymatic hydrolysis, leading to reduced cellulase dosage for the hydrolysis of lignocellulosic biomasses (Borjesson et al., 2007; Kumar and Wyman, 2009; Yang et al., 2011). Different explanations to the surfactant effect on cellulose hydrolysis have been proposed until now, including the effect surfactants could have on enzyme–substrate interactions, limiting the non-productive adsorption of enzymes on the substrate, as well as their ability to stabilize the surface tension in a solution, thus increasing enzyme stability and prevent denaturation of enzymes during hydrolysis (Eriksson et al., 2002a; Yang et al., 2011; Feng et al., 2013; Okino et al., 2013). Triton X-100 that was used as an additive to the hydrolysis experiments described in this study is a non-ionic surfactant that has a hydrophilic polyethylene oxide chain an aromatic hydrocarbon group. Although, together with Tween, Triton surfactants have showed the best improvements of lignocellulose conversion, they are not suitable for large-scale use because of the environmental effects due to the presence of the aromatic ring in the surfactant. Biosurfactants, surface-active substances synthesized by living cells, are becoming more and more popular for their high efficiency and virulence (Feng et al., 2013).

Accessory enzymes (LPMOs, xylanases, and feruloyl-esterases) can all significantly enhance the hydrolytic performance of cellulase enzyme mixtures. The recently discovered family of AA9 LPMOs includes metallo-enzymes that cleave cellulose chain by an oxidative mechanism with the reaction taking place at the protein's active site through a divalent metal ion, *a type II copper*, and, uniquely, a methylated histidine in the copper's coordination sphere (Quinlan et al., 2011; Aachmann et al., 2012; Li et al., 2012). For efficient performance of these enzymes, a reductant/redox-active cofactor that works as an external electron donor is needed (Forsberg et al., 2011). Cannella et al. (2012) showed that under commercially relevant conditions, around 4.1% of the glycosidic bonds in cellulose were oxidatively cleaved by presumably LPMO enzymes. In this study, the addition of *Mt*GH61 resulted in a significant increase of Glc yield; however, to detect the total extent of enhancement, determination of oxidative products should also be conducted. It has been demonstrated that non-cellulosic material present in the pretreated substrates, such as lignin and hemicellulose, is able to act as a cofactor providing reductant residues for the LPMO activity, so as no additional reducing agent is required (Dimarogona et al., 2012; Hu et al., 2014; Westereng et al., 2015). The synergistic interaction of xylanase with cellulases during hydrolysis of the cellulosic component of biomass has been demonstrated in earlier studies using corn stover (Alvira et al., 2011; Qing and Wyman, 2011). The observed xylanase-boosting effect is attributed to the removal of hemicellulose that has remained associated with the cellulosic-rich water insoluble fraction after pretreatment (Chandra et al., 2007). Xylanases have also been shown to result in the solubilization of lignin fractions from pretreated lignocellulosic biomass by breaking down the lignin-carbohydrate complex and consequently improving substrate digestibility (deJong et al., 1997; Suurnakki et al., 1997). In a way similar to EGs, they affect the viscosity of the reaction mixture and facilitate the substrate liquefaction by either increasing the free water in the hydrolysis system or reducing particle size of the pretreated biomass (Viamajala et al., 2009; Di Risio et al., 2011). Hu et al. (2015) has shown that replacing some of the cellulase mixture with an equivalent amount of xylanase leads to increased hydrolysis of the pretreated corn stover substrate substantially, by about 30% and poplar by 8%. In the present study, it was apparent that xylanase treatment could significantly improve the cellulose hydrolysis of all of the lignocellulosic substrates assessed, even for the steam pretreated softwood that contained virtually no xylan. Moreover, it has been suggested that xylanases, apart from the cleavage of hemicellulolytic bonds, they also interact with cellulases by altering gross fiber characteristics, like fiber swelling and fiber porosity. In that way, cellulose accessibility is increased and hydrolysis yields rise (Hu et al., 2011). In a similar way, LPMOs and carbohydrate-binding modules, CBMs have been suggested to promote the amorphogenesis of substrates, thus enhancing the effectiveness of cellulase enzymes (Arantes and Saddler, 2011). A synergistic effect between cellulases, FAEs and xylanases for the hydrolysis of wheat straw has been proven (Tabka et al., 2006; Selig et al., 2008). Ferulic acid is the most abundant hydroxy cinnamic acid in the cell wall (Mueller-Harvey and Hartley, 1986) and is covalently cross-linked to monocot and hardwood arabinoxylans by ester bonds and to components of lignin mainly by ether bonds (Akin et al., 1996). The lignin monomers, monolignols, also with help of ferulic acid, cross-link between each other and with hemicelluloses and increase the density of the cell wall. In case of pretreated spruce, where lignin reaches 47% of the total biomass, incorporation of feruloyl-esterase in the enzyme mixture is imperative. Accessory enzymes such as feruloyl esterases should also act in synergy with xylanases by cleaving diferulic bridges between xylan chains, opening the structures, releasing lignin and increase the accessibility of cellulases to the substrate (Faulds and Williamson, 1995; Yu et al., 2002).

Hydrolysis ratios of this tailor-made enzyme cocktail can be improved with the addition of other enzyme components. It was shown in the literature that xylanases from different families (10 and 11) act synergistically leading to increase of Glc yields (Banerjee et al., 2010a,b; Gao et al., 2011; Zhang et al., 2011). In our experiments we used one xylanase from *M. thermophila*, at concentration of 2–3% of the total enzyme loading. The required amount of accessory enzymes and the extent of their synergistic cooperation with cellulases has been shown to be highly substrate, dependent during hydrolysis of pretreated lignocellulosic substrates (Hu et al., 2015). Though the addition of enzymes with xylanolytic activity would lead only to minor improvements on steam-exploded wheat straw, as this substrate contains only very little xylan, it can be hypothesized that an additional xylanase (as well as β-xylosidase) would raise the release of reducing sugars from birch (xylan is the dominant hemicelluloses component) and other forest materials.

## Conclusion

The efficient hydrolysis of pretreated lignocellulosic substrates requires a consortium of enzymes mainly comprised of high levels of cellulases, together with lower amounts of enzymes that attack non-cellulosic components, such as lignin and hemicellulose. Attempts to improve the performance of such enzyme complexes have mainly focused on cellulases because cellulose is the dominant component in lignocellulose, as well as the main source of glucose for the production of energy, chemicals and materials. As a first step toward evaluating this approach for improving the enzymatic cocktails, the increase of the enzymatic hydrolysis of different agricultural and forest residues, such as wheat straw, softwoods (spruce), and hardwoods (birch) was investigated. These substrates show significant quantitative and qualitative differences in their non-cellulosic polysaccharide components. A statistical model was set up to search for optimized enzymatic mixtures containing four core enzymes, in the presence of other five “accessory” enzymes, all encoded by *M. thermophila*'s genes. The present results suggest that *Mt*CBH7 and *Mt*EG7 are enzymes of major importance for optimized final reducing sugars and glucose yields during the hydrolysis of pretreated wheat straw, while *Mt*CBH7 plays a crucial role in case of spruce. CBHs *Mt*CBH6 and *Mt*CBH7 act in combination and are key enzymes for the hydrolysis of the hardwood (birch). For the hydrolysis of the pure substrate (PASC), high proportions of endoglucanases, especially *Mt*EG7 are needed for efficient yields.

## Author contributions

AK produced the enzymes, carried out the hydrolysis experiments and wrote the manuscript. LM set up the experimental design and developed the statistical models. ET contributed in the molecular cloning and heterologous expression of the enzymes. UR and PC participated in study conception, data interpretation and corrected the manuscript. All authors have read and approved the final manuscript.

### Conflict of interest statement

The authors declare that the research was conducted in the absence of any commercial or financial relationships that could be construed as a potential conflict of interest.
