# Supplementary material for: Development of Thermophilic Tailor-Made Enzyme Mixtures for the Bioconversion of Agricultural and Forest Residues
Source: Front Microbiol. 2016 Feb 16;7:177. doi: 10.3389/fmicb.2016.00177 (PMC4754399; doi:10.3389/fmicb.2016.00177)
Supplement: Supplementary file 1 [file DataSheet1.DOC]

**“Development of thermophilic tailor-made enzyme mixtures for the bioconversion of agricultural and forest residues”**

Anthi Karnaouri^1^, Leonidas Matsakas^1^, Evangelos Topakas^1,2^, Ulrika Rova^1^, Paul Christakopoulos^1*^

**Supplementary Material**

**
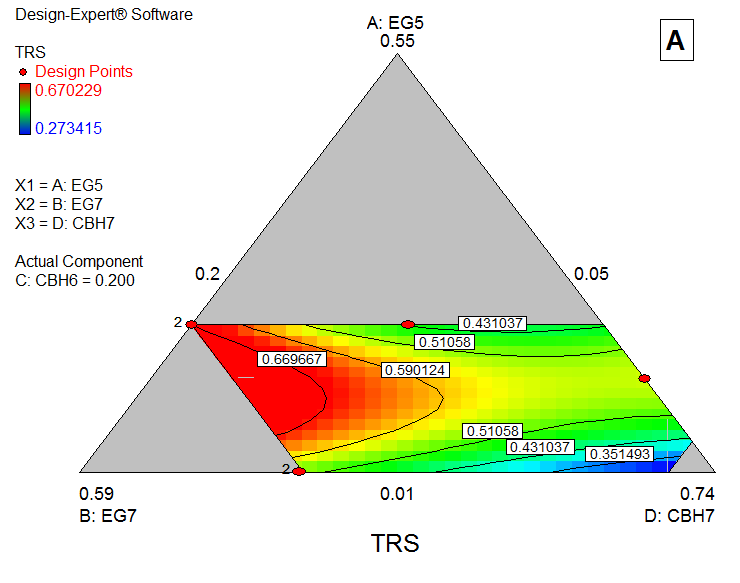

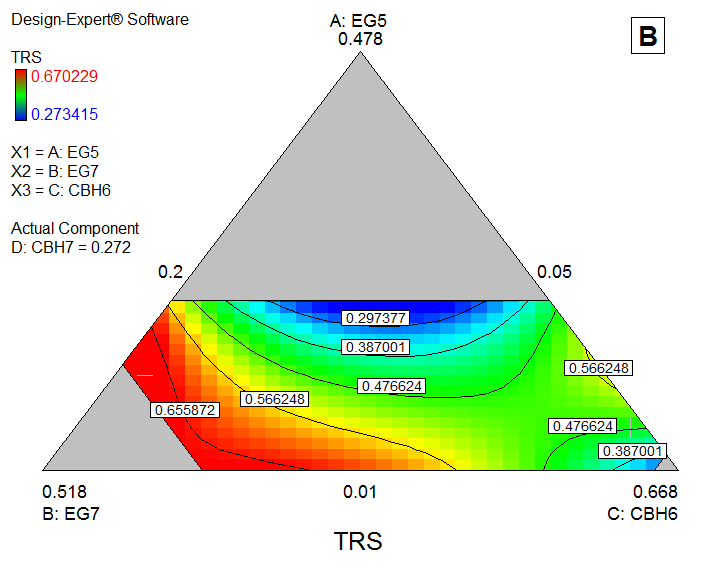
**


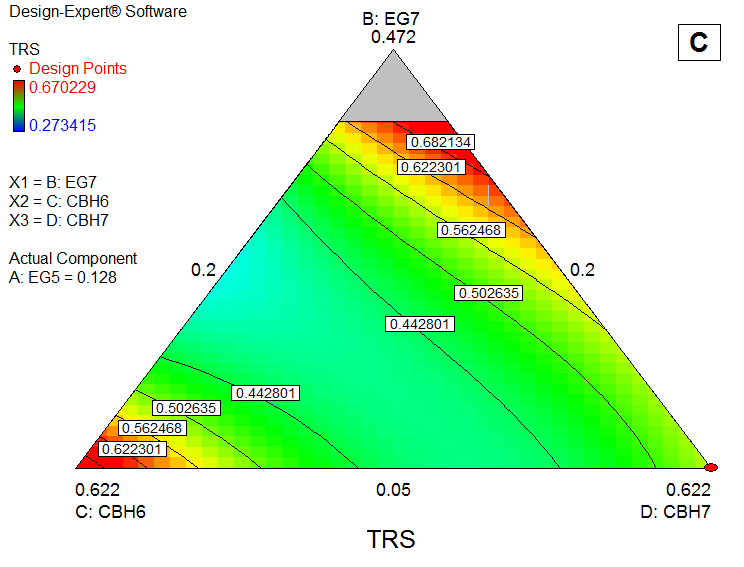


**
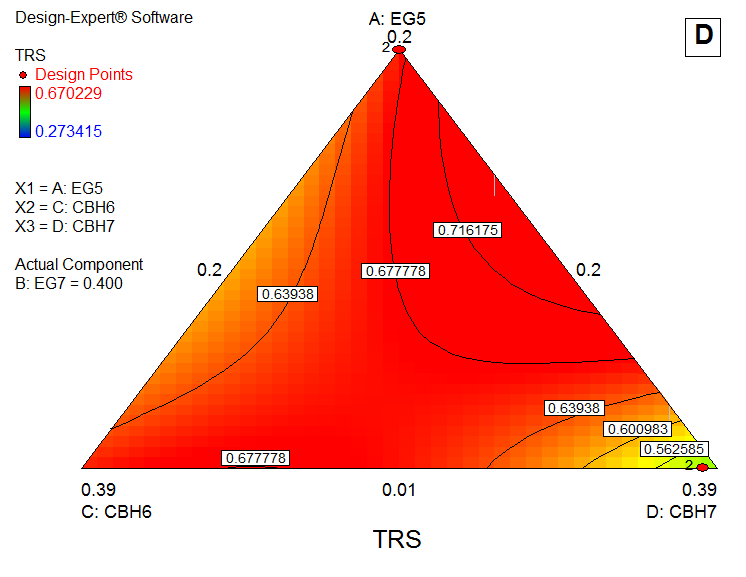
**

**Fig.1.** Ternary plots showing predicted final **TRS** yields from *PASC* hydrolysis, as a function of three out of four “core” enzymes content (X1, X2, and X3). For each plot, the forth enzyme (“actual component”) has been fixed to the proportion of the point resulting in the optimal sugar yield, as predicted by the model.

**
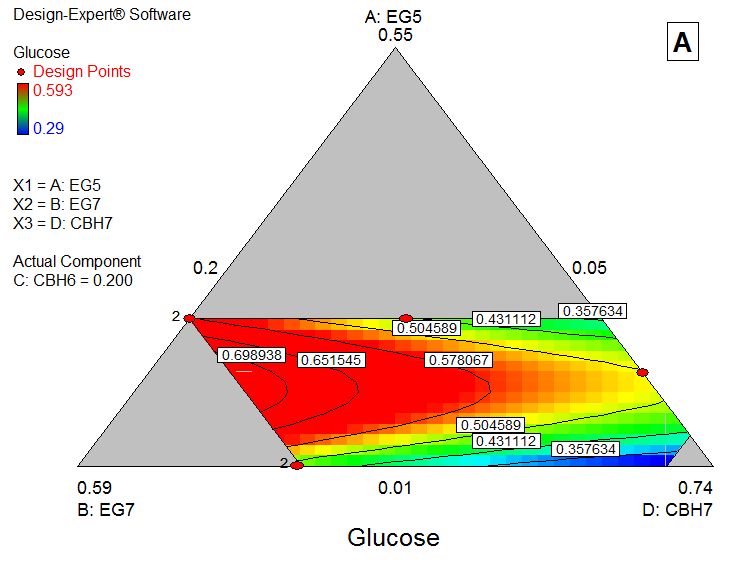

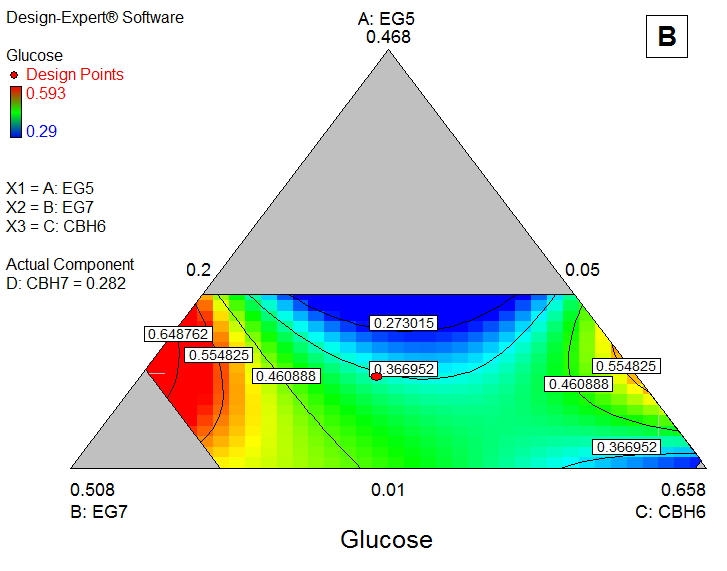

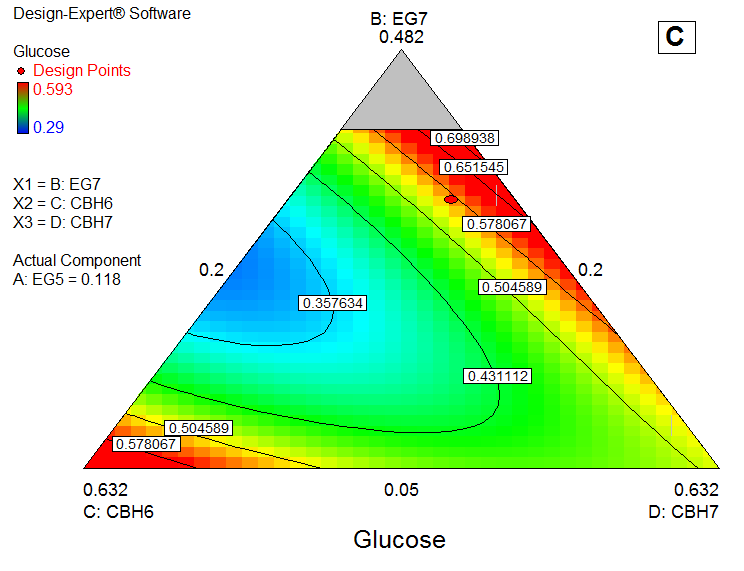

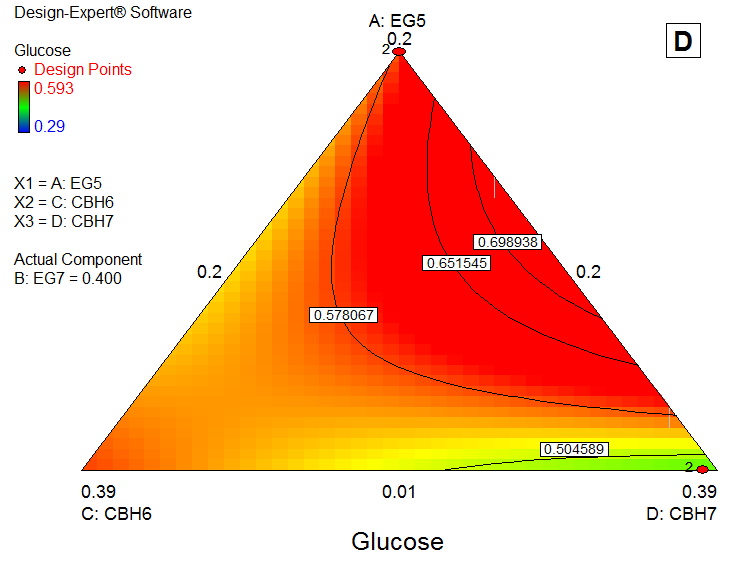
**

**Fig.2.** Ternary plots showing predicted final **Glc** yields from *PASC* hydrolysis, as a function of three out of four “core” enzymes content (X1, X2, and X3). For each plot, the forth enzyme (“actual component”) has been fixed to the proportion of the point resulting in the optimal sugar yield, as predicted by the model.

**
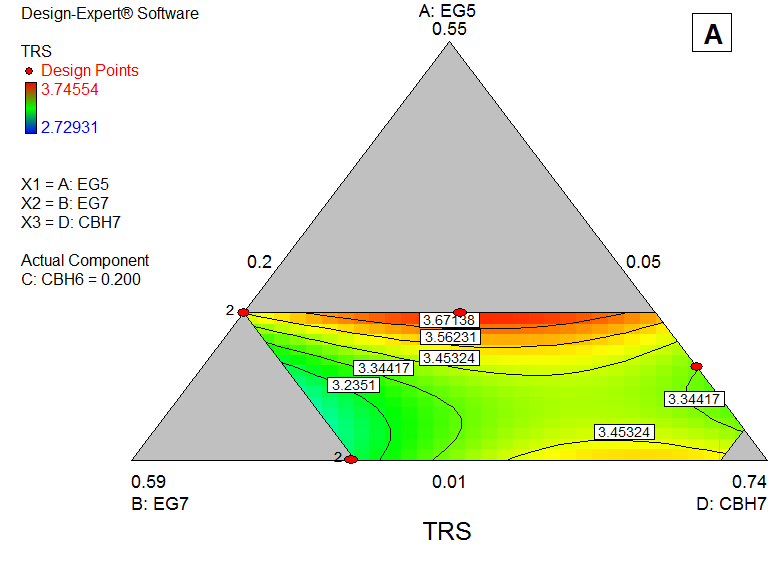

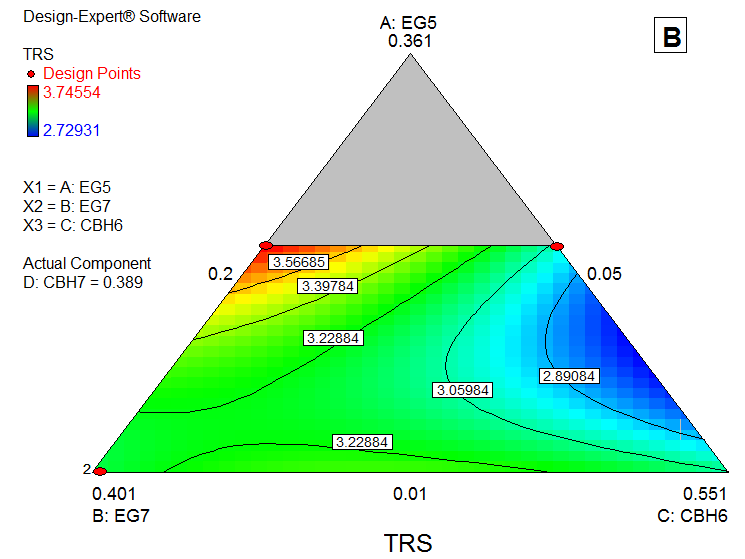
**

**
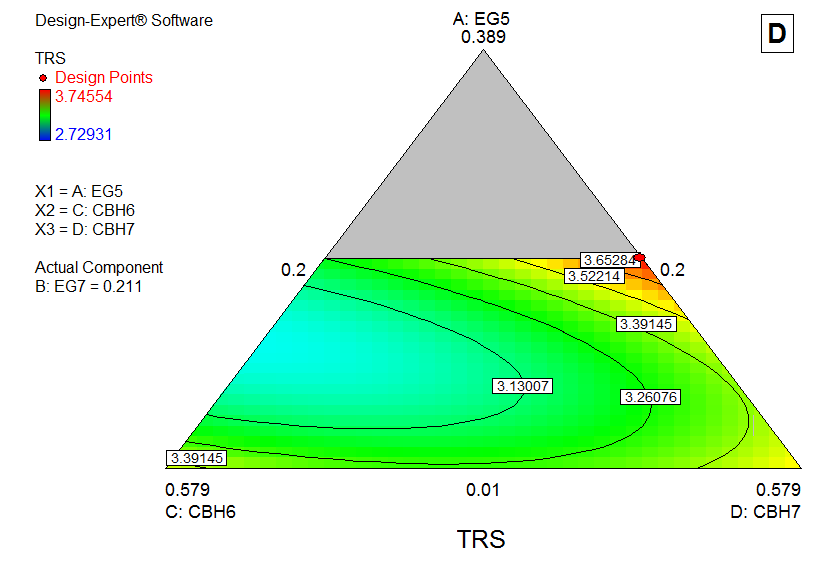
**

**
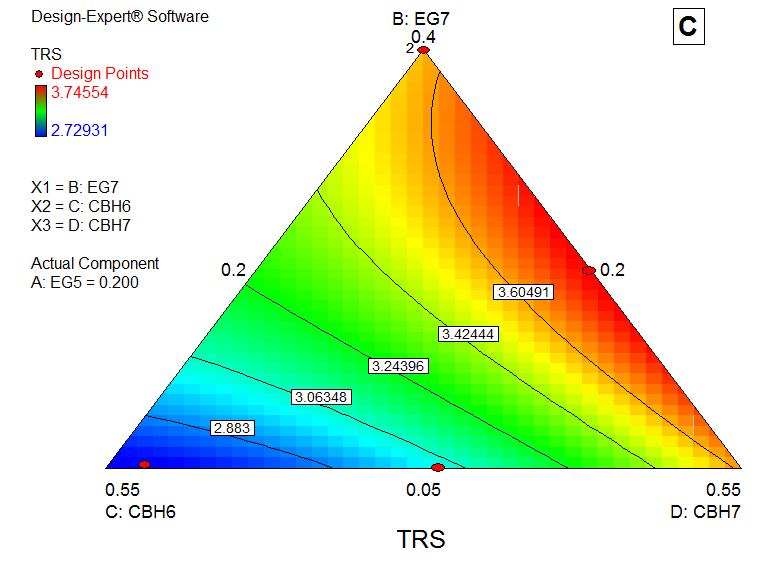
**

**Fig.3.** Ternary plots showing predicted final **TRS** yields from *wheat straw* hydrolysis, as a function of three out of four “core” enzymes content (X1, X2, and X3). For each plot, the forth enzyme (“actual component”) has been fixed to the proportion of the point resulting in the optimal sugar yield, as predicted by the model.

**
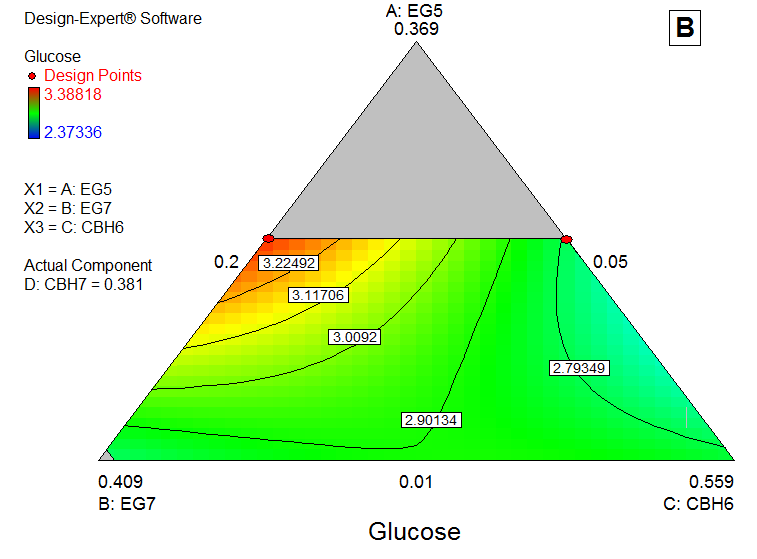
**

**
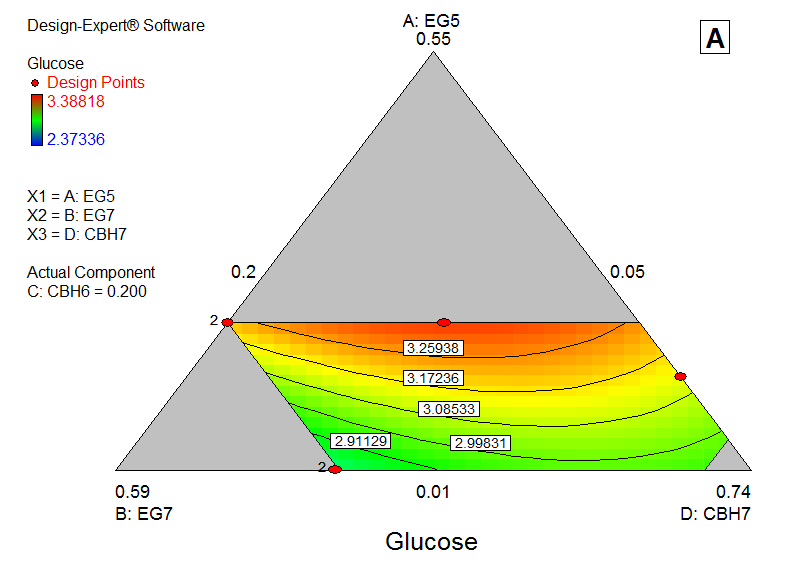
**

**
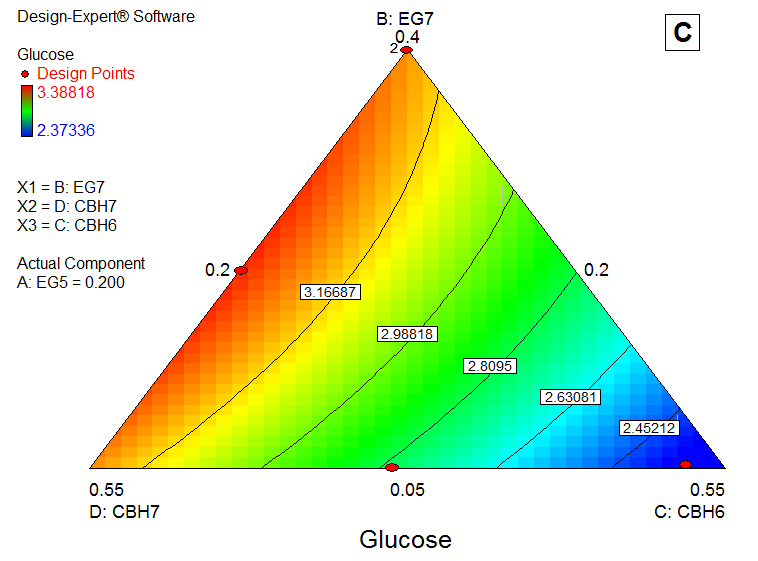

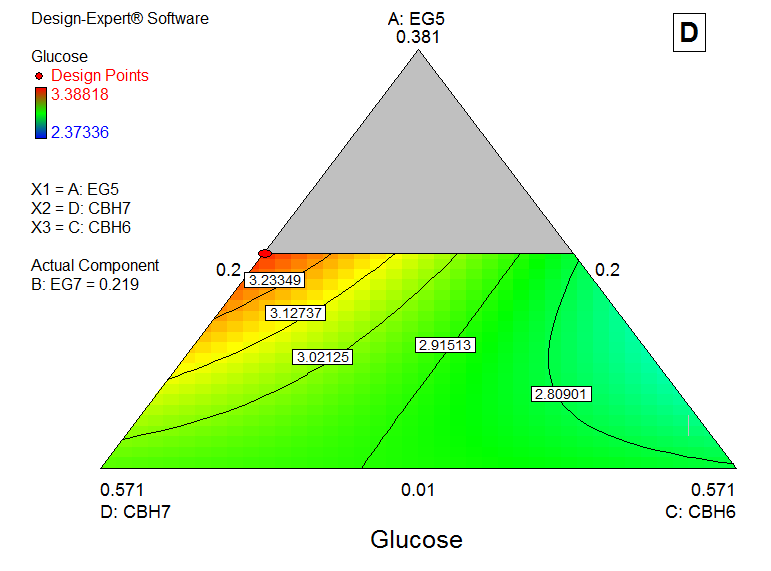
**

**Fig.4.** Ternary plots showing predicted final **Glc** yields from *wheat straw* hydrolysis, as a function of three out of four “core” enzymes content (X1, X2, and X3). For each plot, the forth enzyme (“actual component”) has been fixed to the proportion of the point resulting in the optimal sugar yield, as predicted by the model.

**
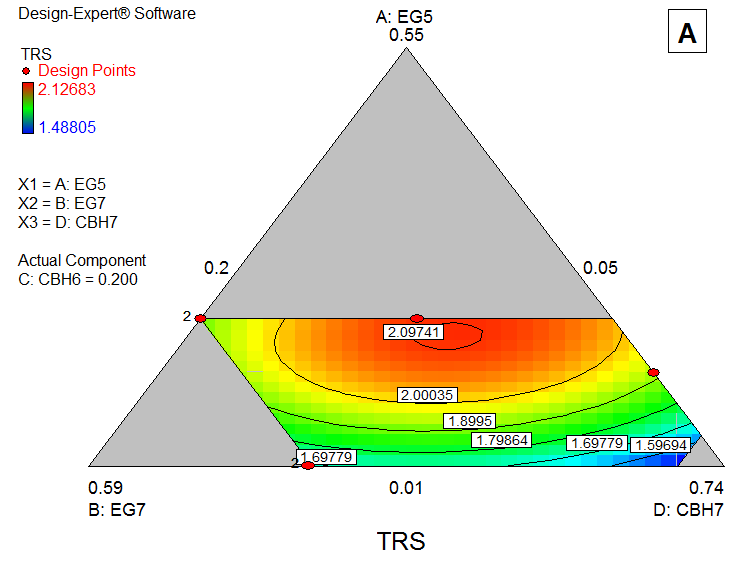

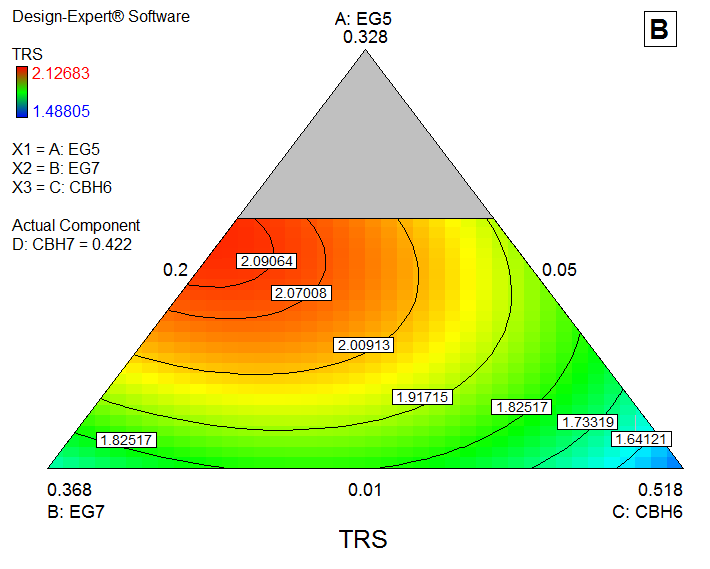
**

**
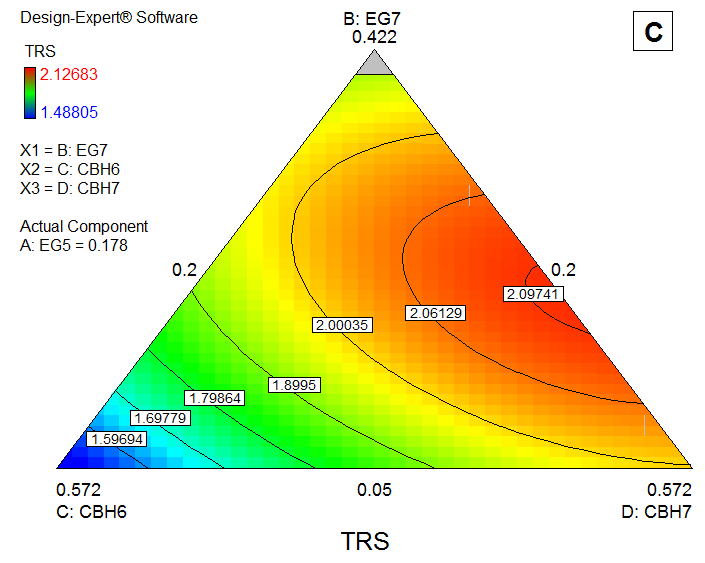

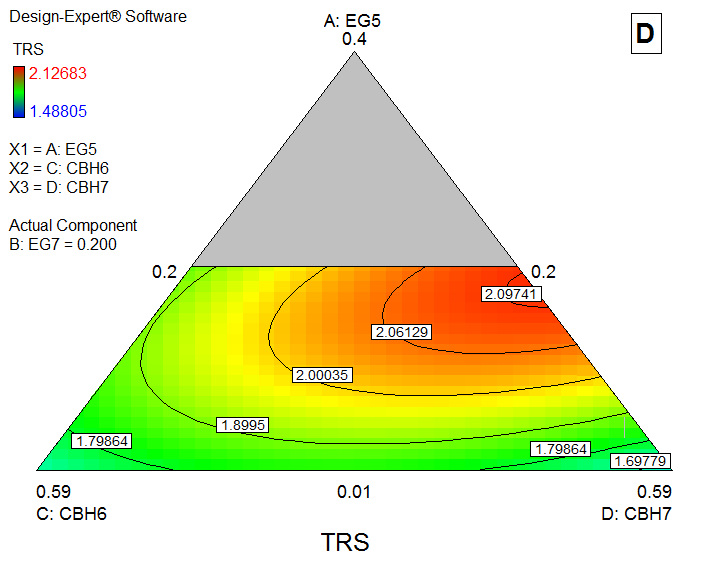
**

**Fig.5.** Ternary plots showing predicted final **TRS** yields from *spruce* hydrolysis, as a function of three out of four “core” enzymes content (X1, X2, and X3). For each plot, the forth enzyme (“actual component”) has been fixed to the proportion of the point resulting in the optimal sugar yield, as predicted by the model.

**
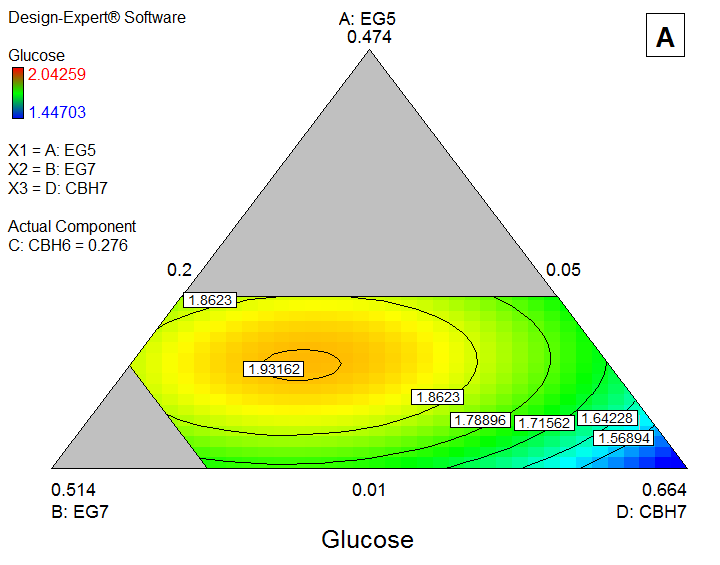

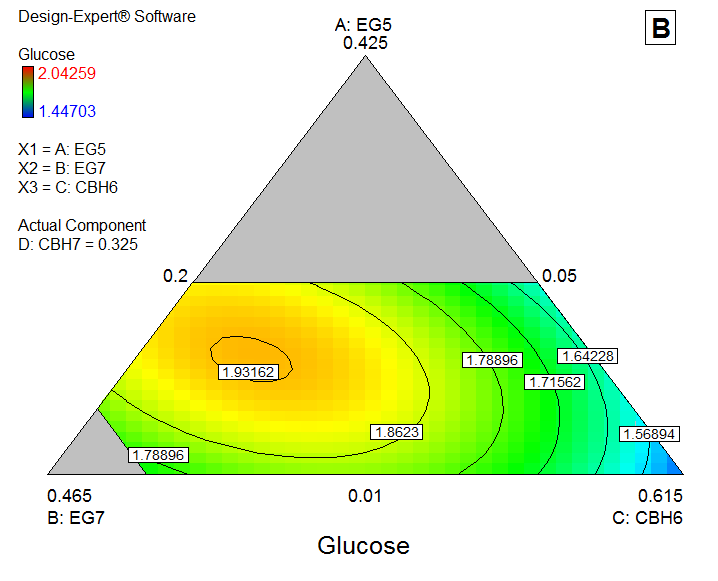
**

**
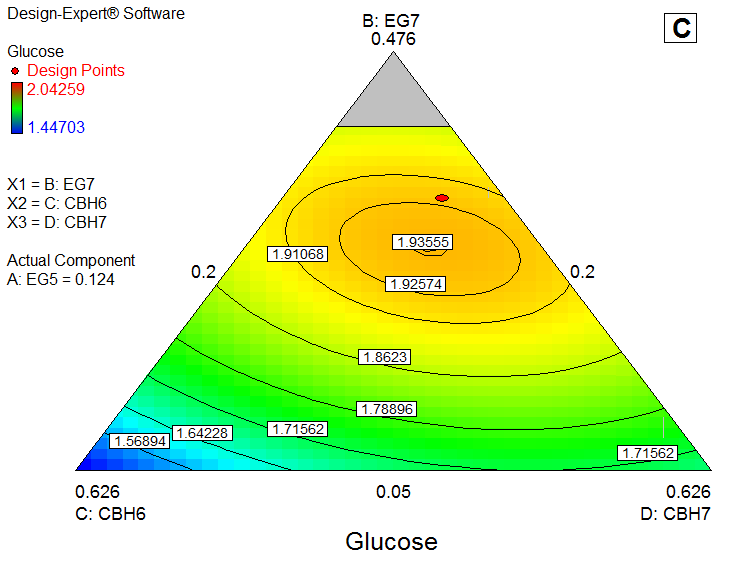

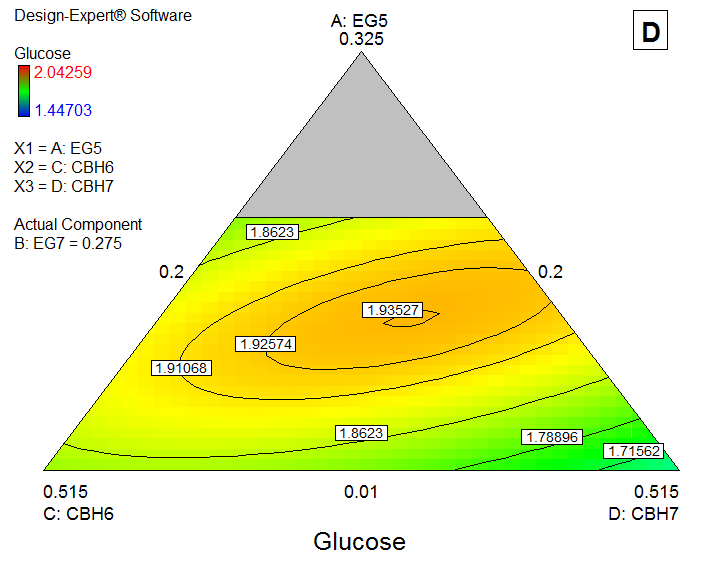
**

**Fig.6.** Ternary plots showing predicted final **Glc** yields from *spruce* hydrolysis, as a function of three out of four “core” enzymes content (X1, X2, and X3). For each plot, the forth enzyme (“actual component”) has been fixed to the proportion of the point resulting in the optimal sugar yield, as predicted by the model.

**
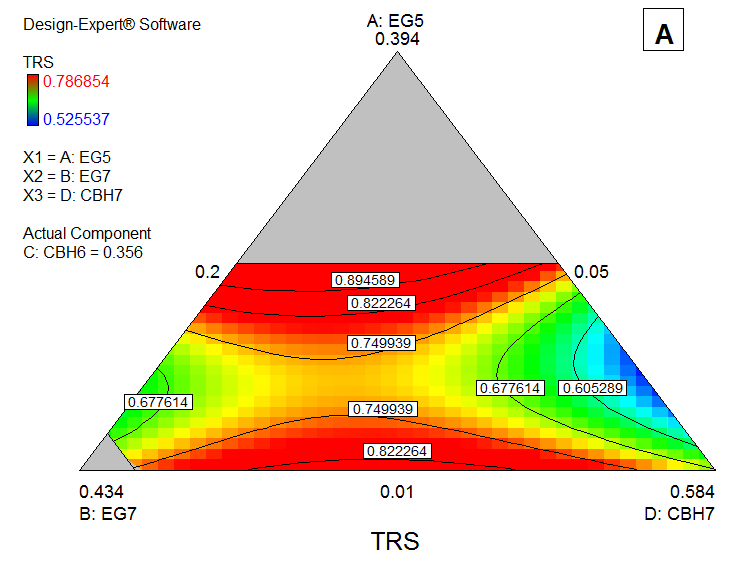

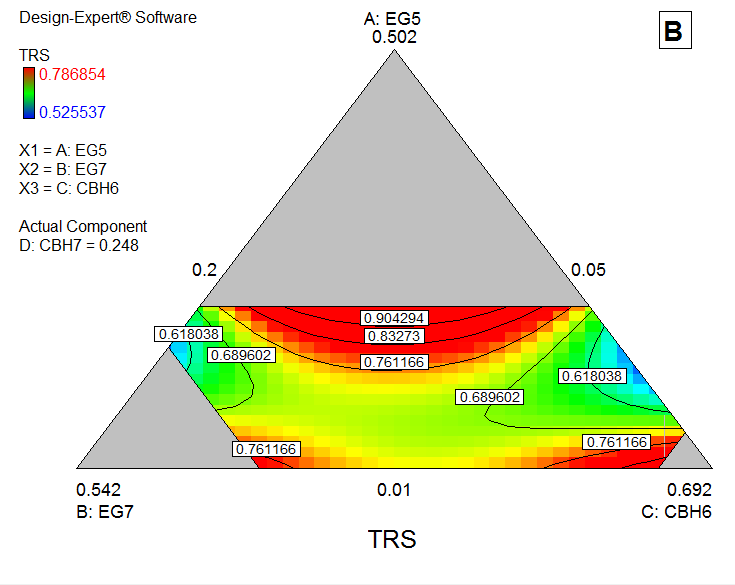
**

**
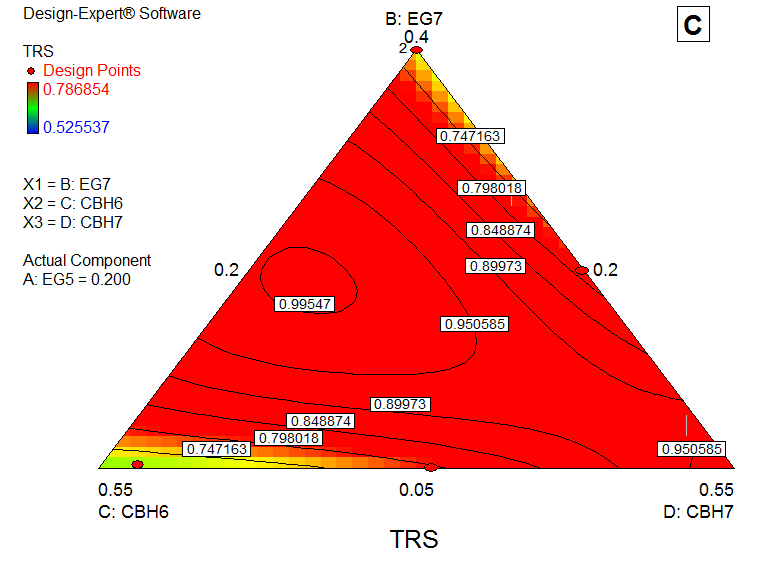

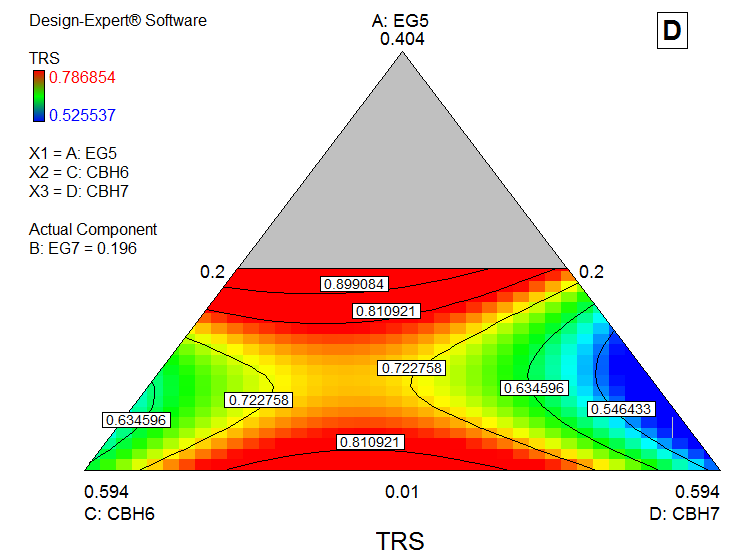
**

**Fig.7.** Ternary plots showing predicted final **TRS** yields from *birch* hydrolysis, as a function of three out of four “core” enzymes content (X1, X2, and X3). For each plot, the forth enzyme (“actual component”) has been fixed to the proportion of the point resulting in the optimal sugar yield, as predicted by the model.

**
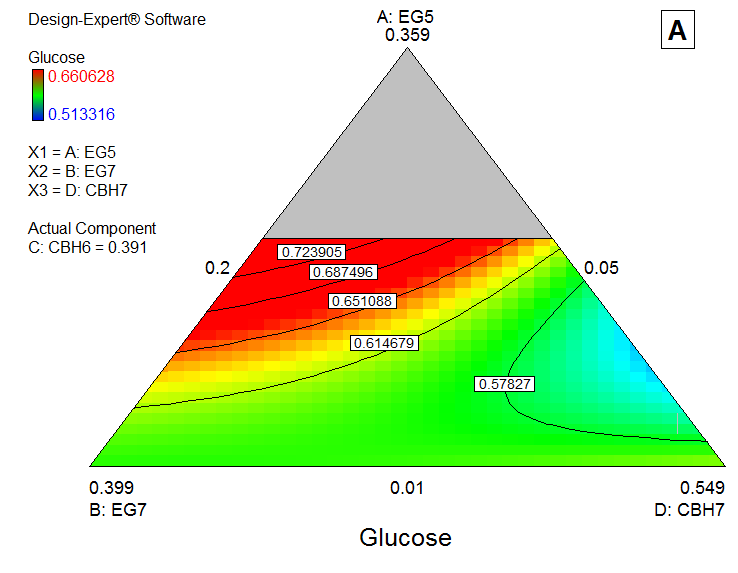

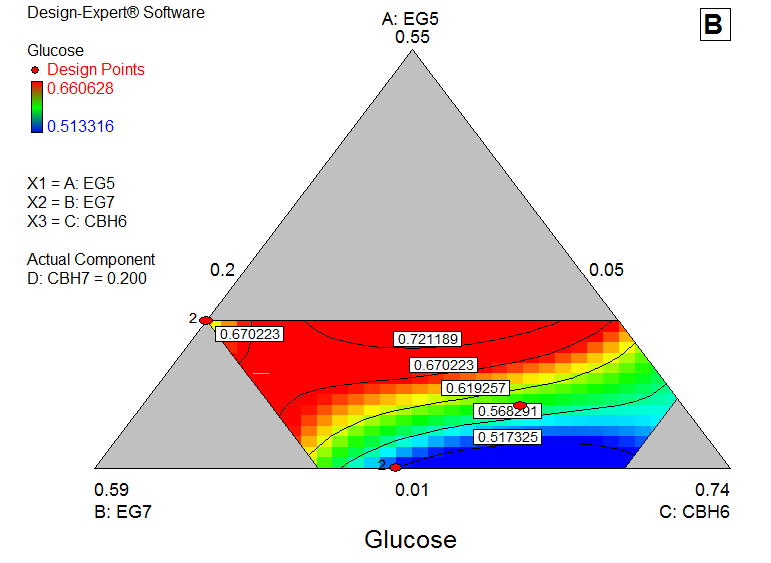

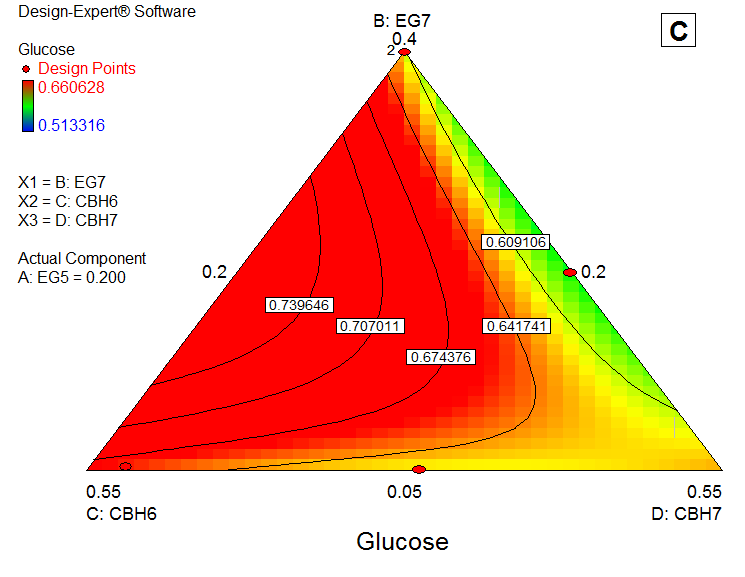

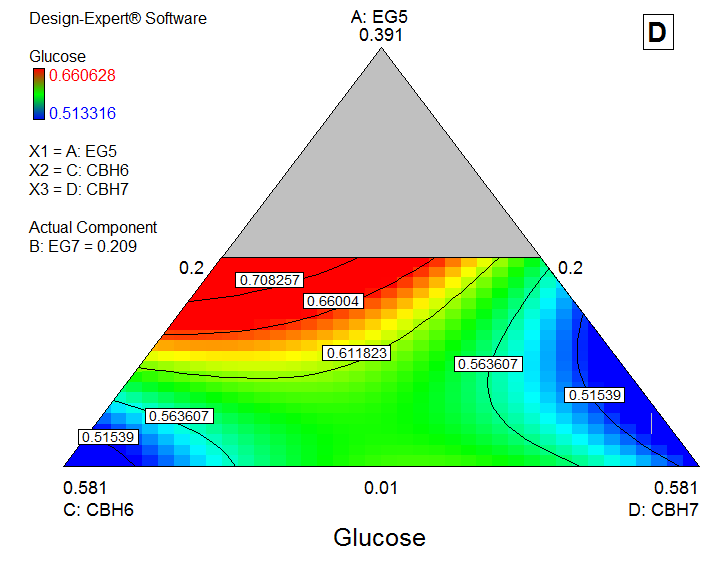
**

**Fig.8.** Ternary plots showing predicted final **Glc** yields from *birch* hydrolysis, as a function of three out of four “core” enzymes content (X1, X2, and X3). For each plot, the forth enzyme (“actual component”) has been fixed to the proportion of the point resulting in the optimal sugar yield, as predicted by the model.

**Table 1.** Yields from *PASC* hydrolysis, 48h of incubation.

**Table 2.** Yields from *wheat straw* hydrolysis, 48h of incubation.

**Table 3.** Yields from *spruce* hydrolysis, 48h of incubation.

**Table 4.** Yields from *birch* hydrolysis, 48h of incubation.
